# Supplementary material for: The Spindle Assembly Checkpoint Is Not Essential for Viability of Human Cells with Genetically Lowered APC/C Activity
Source: Cell Rep. 2016 Feb 18;14(8):1829–40. doi: 10.1016/j.celrep.2016.01.060 (PMC4785794; doi:10.1016/j.celrep.2016.01.060)
Supplement: Document S1. Supplemental Experimental Procedures and Figures S1–S5 [file mmc1.pdf]

Cell Reports, Volume *14*

Supplemental Information

The Spindle Assembly Checkpoint Is Not Essential for Viability of Human Cells with Genetically Lowered APC/C Activity

Thomas Wild, Marie Sofie Yoo Larsen, Takeo Narita, Julie Schou, Jakob Nilsson, and Chunaram Choudhary

Supplemental Information

The spindle assembly checkpoint is not essential for viability of human cells with genetically lowered APC/C activity

Thomas Wild[1,*], Marie Sofie Yoo Larsen[2,*], Takeo Narita[1], Julie Schou[2], Jakob Nilsson[2,§], Chunaram Choudhary[1,§]

[1]Proteomics Program, the Novo Nordisk Foundation Center for Protein Research, Faculty of Health and Medical Sciences, University of Copenhagen, Blegdamsvej 3B, DK-2200 Copenhagen, Denmark
[2]Protein Signaling Program, the Novo Nordisk Foundation Center for Protein Research, Faculty of Health and Medical Sciences, University of Copenhagen, Blegdamsvej 3B, DK-2200 Copenhagen, Denmark

*These authors contributed equally to this work.

[§]Correspondence should be addressed to: jakob.nilsson@cpr.ku.dk, or chuna.choudhary@cpr.ku.dk

Supplemental figures

Figure S1

A

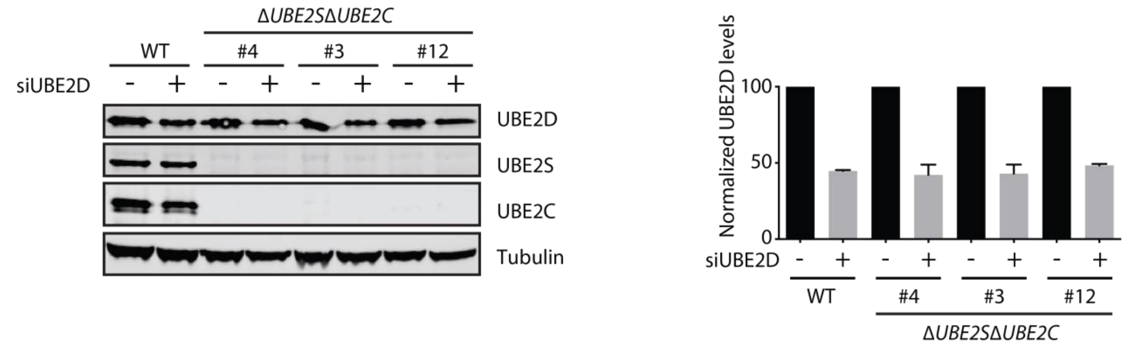

B

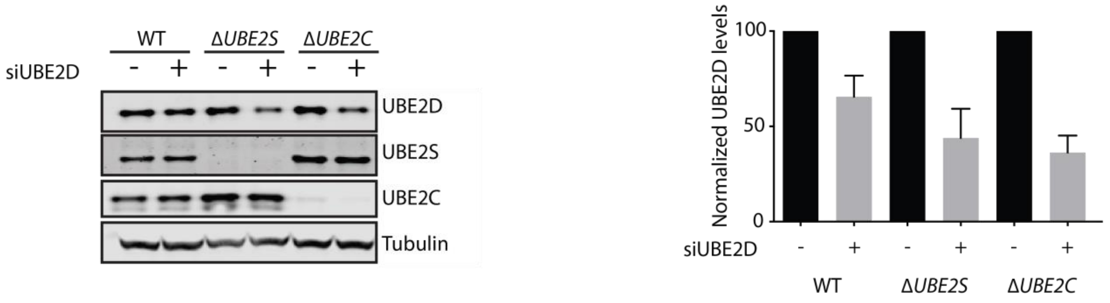

Figure S1, related to Figure 2. Validation of UBE2D depletion by RNAi.

- **A.** The left panel shows immunoblot-based validation of *UBE2D* knockdown in RNAi experiment shown in **Figure 2D**. Results from a representative experiment are shown. The bar chart (the right panel) shows *UBE2D* knockdown efficiency from two independent experiments. For each cell line, the normalized UBE2D levels show the ratio of UBE2D intensity over tubulin intensity. This ratio was set to 100% in the control siRNA condition and UBE2D levels in *UBE2D* siRNA-treated cells are shown relative to the control treated cells.
- **B.** Confirmation of *UBE2D* knockdown for the data shown in **Figure 2E**. The immunoblot (the left panel) shows results from a representative experiment. The bar chart (the right panel) shows knockdown efficiency from three independent experiments, the percent of UBE2D knockdown was determined as described in **Figure S1A**.

Figure S2

A

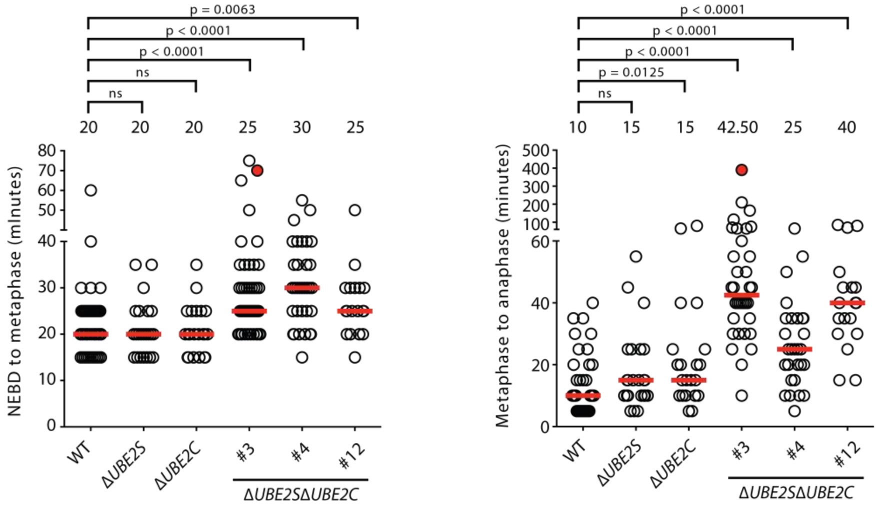
p = 0.0063
p < 0.0001
p < 0.0001
ns
ns
20 20 20 25 30 25
NEBD to metaphase (mInutes)
WT
ΔUBE2S
ΔUBE2C
#3
#4
#12
ΔUBE2SΔUBE2C
p < 0.0001
p < 0.0001
p < 0.0001
p = 0.0125
ns
10 15 15 42.50 25 40
Metaphase to anaphase (minutes)
WT
ΔUBE2S
ΔUBE2C
#3
#4
#12
ΔUBE2SΔUBE2C

B

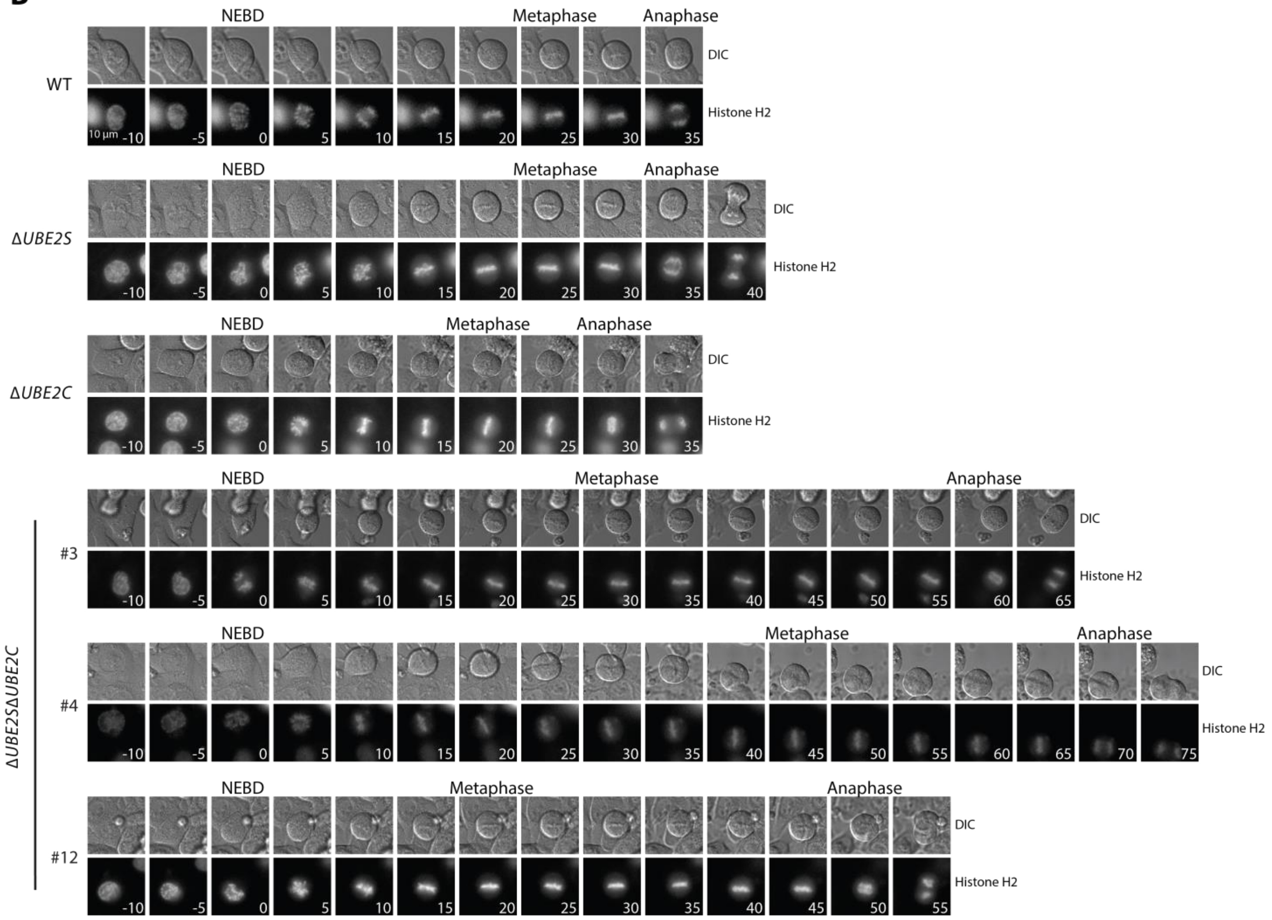
NEBD
Metaphase
Anaphase
WT
DIC
Histone H2
10 µm
ΔUBE2S
ΔUBE2C
#3
#4
#12
ΔUBE2SΔUBE2C

Figure S2, related to Figure 2. Mitotic timing in ∆*UBE2S*, ∆*UBE2C*, and ∆*UBE2S*∆*UBE2C* cells.

A. Timing for NEBD to metaphase (left) and metaphase to anaphase onset (right) for the indicated cell lines. Cells were transiently transfected with histone H2B-RFP and analyzed by time-lapse microscopy. Each circle represents a single cell (red-filled circles indicate cells that did not exit mitosis within the stated time). The median time is depicted by a red line and noted on the top of the data points. For each cell line, at least 18 cells were analyzed from at least two independent experiments. P values were calculated with Mann-Whitney tests. ns = $p \geq 0.01$.

B. Representative still images from live cell imaging experiment shown in **Figure S2A**. For ∆*UBE2S*∆*UBE2C* cells data are shown from three different cell clones.

Figure S3

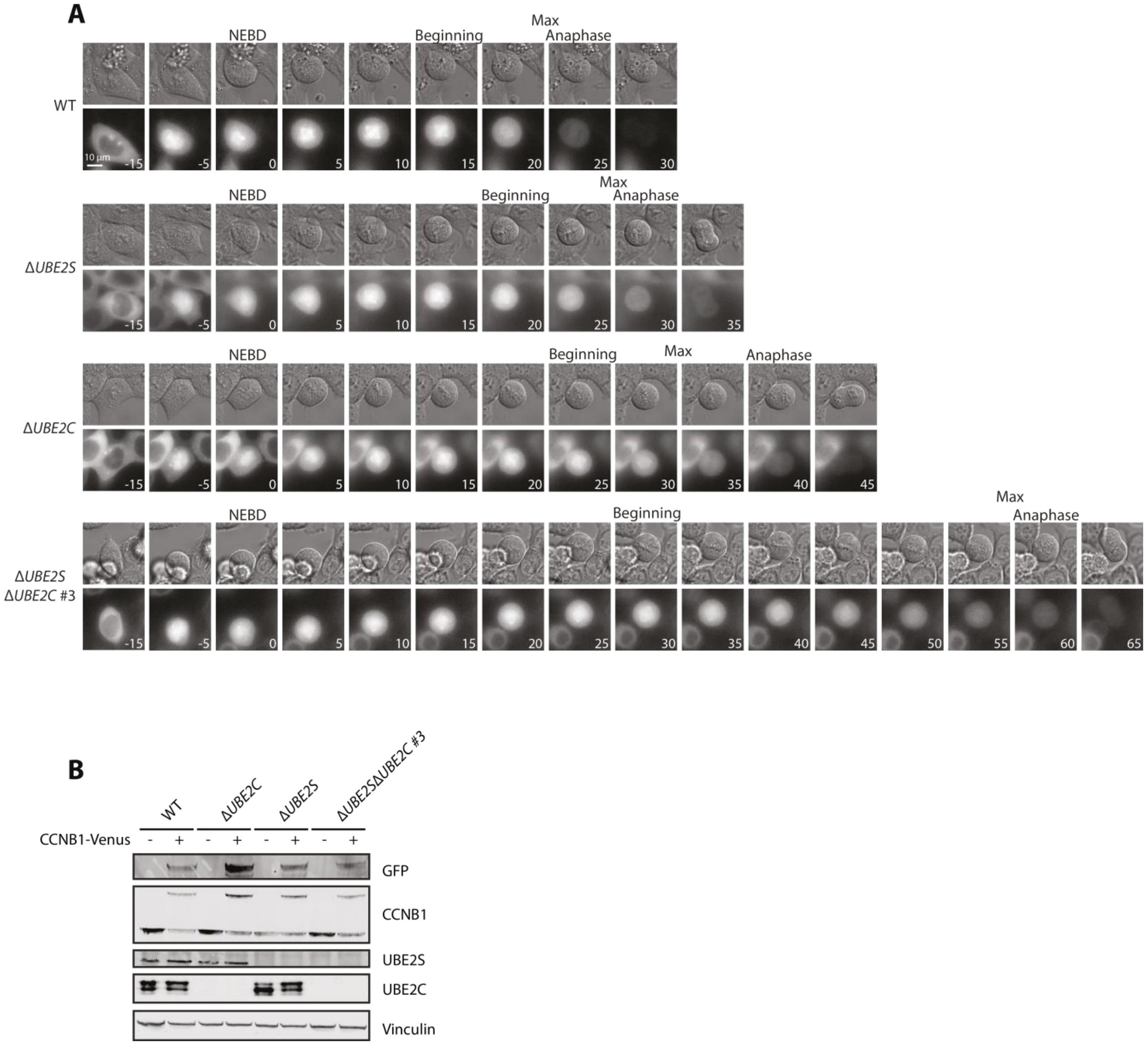

Figure S3, related to Figure 2F,G. CCNB1 degradation in Δ*UBE2S*, Δ*UBE2C*, and Δ*UBE2S*Δ*UBE2C* cells.

- **A.** Representative still images from live cell imaging experiment shown in **Figure 2F**. Frames marking the beginning and maximal (max) CCNB1 degradation are labeled accordingly.
- **B.** Western blot analysis of the indicated proteins in the analyzed cells. The figure confirms the expression of CCNB1-Venus (detected using a GFP antibody that also recognize Venus, and using a CCNB1 antibody). UBE2S and UBE2C immunoblots confirm the absence of these proteins in the respective knockout cells, and vinculin is shown as loading control.

Figure S4

A

Cell line	Reversine	R squared	LDT (h)	95 % CI LDT	Intercept (ln)	95 % CI ln	AUC	95 % CI AUC
WT	-	0.99	21.8	21.2 - 22.5	14.1	14.0 - 14.2	104.5	104.0 - 105.1
ΔUBE2S	-	0.99	24.0	23.3 - 24.8	14.5	14.3 - 14.6	104.8	104.5 - 105.1
ΔUBE2C	-	0.99	23.5	22.6 - 24.4	14.5	14.3 - 14.6	105.3	104.8 - 105.8
ΔUBE2SΔUBE2C #3	-	0.95	37.0	34.1 - 40.4	14.4	14.2 - 14.6	98.2	97.5 - 99.0
ΔUBE2SΔUBE2C #3	+	0.88	60.3	53.1 - 69.8	14.5	14.2 - 14.7	93.9	93.3 - 94.6
ΔUBE2SΔUBE2C #4	-	0.96	34.8	32.5 - 37.4	14.5	14.3 - 14.6	99.3	98.7 - 99.9
ΔUBE2SΔUBE2C #4	+	0.95	48.9	45.1 - 53.4	14.4	14.3 - 14.5	95.3	94.8 - 95.8
ΔUBE2SΔUBE2C #8	-	0.99	33.3	32.4 - 34.3	14.3	14.3 - 14.4	99.0	98.8 - 99.3
ΔUBE2SΔUBE2C #8	+	0.97	41.0	38.5 - 43.9	14.3	14.2 - 14.4	96.4	96.0 - 96.9
ΔUBE2SΔUBE2C #12	-	0.99	32.6	31.6 - 33.7	14.2	14.2 - 14.3	98.7	98.4 - 99.0
ΔUBE2SΔUBE2C #12	+	0.97	46.7	43.7 - 50.1	14.2	14.1 - 14.3	94.4	94.0 - 94.9

B

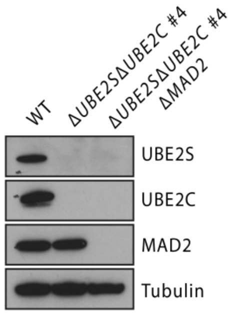

C

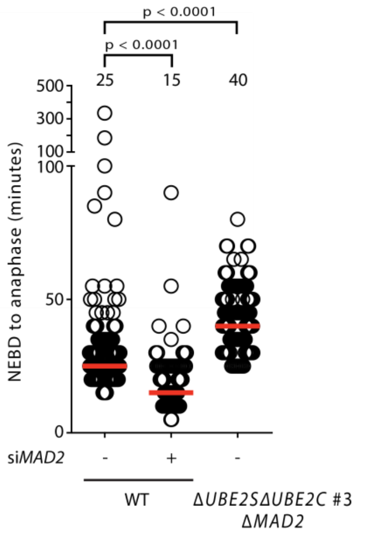

D

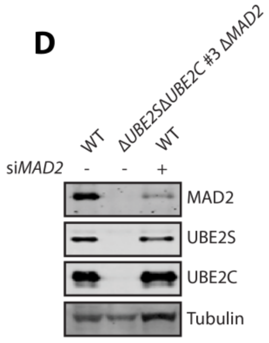

E

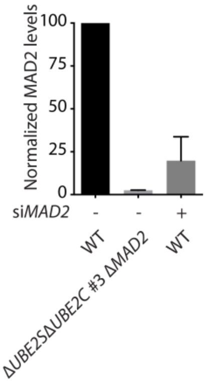

F

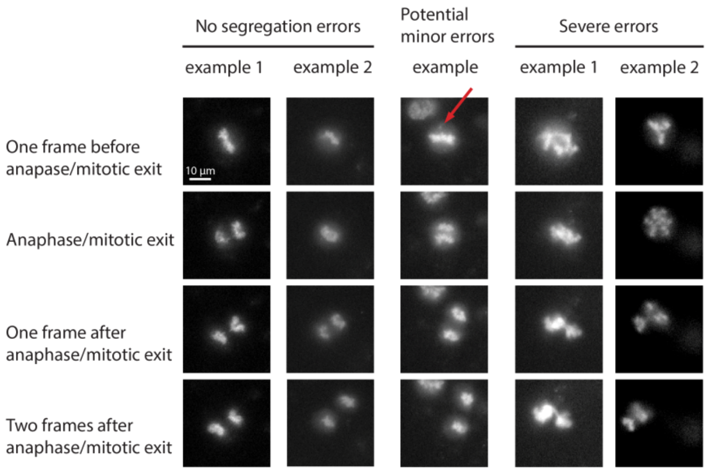

G

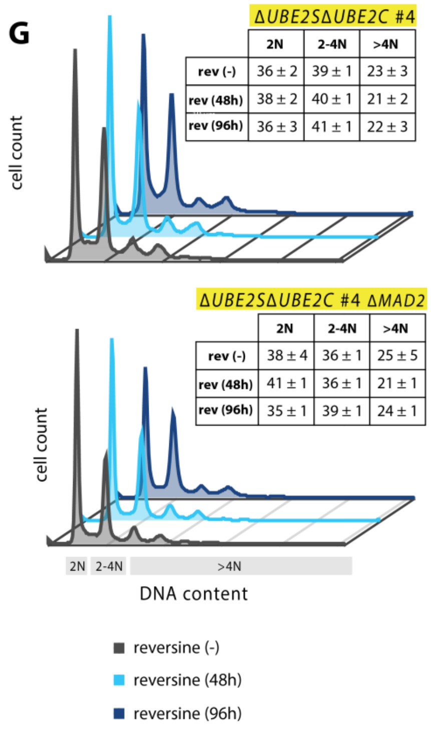

Figure S4, related to Figure 3. The SAC is not essential in Δ*UBE2S*Δ*UBE2C* cells.

- **A.** Calculation of cell doubling time from the data shown in **Figure 3B**. The indicated cell lines were cultured with or without reversine as indicated and cellular ATP content was measured every alternative day for up to 12 days using a luminescence-based assay. A linear regression model for luminescence intensity from day one to day six was used to calculate doubling times for the indicated cell lines and conditions. Cellular ATP content was used as a proxy for calculating cell proliferation; thus, LDT corresponds to cell doubling time. Abbreviations: luminescence doubling time (LDT), confidence interval (CI), intercept (In), area under the curve (AUC).
- **B.** Western blot-based confirmation of MAD2 deletion in Δ*UBE2S*Δ*UBE2C* #4 Δ*MAD2* cells, as control expression of MAD2 is show in the parental Δ*UBE2S*Δ*UBE2C* clone #4 and WT cells.
- **C.** Analysis of NEBD to anaphase onset timing in mitotic cells shown in **Figure 3D.** MAD2 was knocked down using siRNAs in WT cells and their mitotic phenotype was compared to Δ*UBE2S*Δ*UBE2C* #3 Δ*MAD2* cells, in which MAD2 was completely deleted. At least 150 cells were analyzed from two independent experiments. The median is depicted as a red line and noted above the data points. P-values for indicated conditions are stated on top.
- **D.** The immunoblot shows depletion of MAD2 in RNAi-based experiments presented in **Figure 3D**, the blot is representative from one of three experiments.
- **E.** Quantification of MAD2 depletion in RNAi experiments shown in **Figure 3D and Figure S4C**, the data are from three independent experiments.
- **F.** Representative still images from live cell imaging experiments shown in **Figure 3D**. When cells exited mitosis, the segregation of the chromosomes was categorized into three categories: no segregation errors, potential minor errors, or severe errors. The red arrow indicates a potential segregation error.
- **G.** Analysis of cellular DNA content in Δ*UBE2S*Δ*UBE2C* #4 and Δ*UBE2S*Δ*UBE2C* #4 Δ*MAD2* cells upon reversine treatment. The cells were cultured in the indicated conditions and cellular DNA was stained with propidium iodide and cells were analyzed by flow cytometry.

Figure S5

A

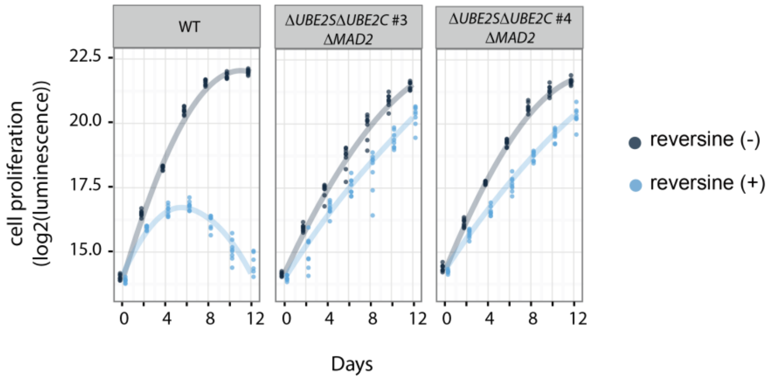

B

Cell line	Reversine	R squared	LDT (h)	95 % CI LDT	Intercept (In)	95 % CI In	AUC	95 % CI AUC
WT	-	0.99	22.6	22.0 - 23.3	14.1	14.0 - 14.3	103.9	103.7 - 104.2
Δ*UBE2S*Δ*UBE2C* #3 Δ*MAD2*	-	0.97	31.9	29.9 - 34.1	14.3	14.1 - 14.4	99.5	98.9 - 100.1
Δ*UBE2S*Δ*UBE2C* #3 Δ*MAD2*	+	0.92	38.9	35.1 - 43.5	14.0	13.8 - 14.2	94.9	94.0 - 95.8
Δ*UBE2S*Δ*UBE2C* #4 Δ*MAD2*	-	0.99	29.6	28.8 - 30.4	14.4	14.4 - 14.5	101.4	101.1 - 101.6
Δ*UBE2S*Δ*UBE2C* #4 Δ*MAD2*	+	0.96	43.3	40.5 - 46.6	14.4	14.3 - 14.5	96.5	96.0 - 96.9

Figure S5, related to Figure 5. Assessment of SAC function in Δ*UBE2S*Δ*UBE2C*Δ*MAD2* cells.

- **A.** Growth analysis of Δ*UBE2S*Δ*UBE2C*Δ*MAD2* cells (clone #3 and #4) in the presence or absence of 0.5 µM reversine, WT cells were used as reference.
- **B.** Doubling times for the indicated cell lines and condition. The doubling time was calculated as described in **Figure S4A**. Abbreviations: luminescence doubling time (LDT), confidence interval (CI), intercept (In), area under the curve (AUC).

Legend for Supplemental Table S1. SILAC-based proteomic analysis of the knockout cells. SILAC-based quantification of changes in protein levels in knockout cells compared to WT cells. The spreadsheet indicates the relative changes in protein expression in the indicated knockout cells compared to WT cells.

Supplemental methods

RNA-mediated interference

Cells were transfected with siRNAs (final concentration of 50 nM) using RNAiMAX (Lipofectamine®) the day before the start of live cell imaging. For depletion of UBE2D, a previously described pool of siRNAs was used (Dynek et al., 2010). For MAD2 depletion, the following siRNA oligo was used: 5'-GGAAGAGUCGGGACCACAG-3'. As a negative control, a siRNA targeting luciferase (Sigma, VC300B2) was used. All siRNAs were obtained from Sigma. To evaluate knockdown efficiencies, the expression of target proteins was analyzed by Western blotting. For quantification, the blots were scanned using the Odyssey Sa imaging system (Li-Cor) and quantification was carried out using the Odyssey Sa Application software (Li-Cor) with IRDye 800 or 680 secondary antibodies (Li-Cor).

Cell proliferation assay

For each cell line and condition, 400 cells were seeded in octuplicate in a 96-well plate. Cells were grown in the absence or presence of reversine (0.5 μM) and the medium was changed every second day. Cell viability was assessed at different time points (day 0 = 12 hours after seeding, before addition of reversine) using the CellTiter-Glo® Luminescent Cell Viability Assay (Promega) according to the manufacturer`s protocol, measuring luminescence with a Varioskan Flash (Thermo Scientific). The local regression curves were plotted using geom_line() function in R package "ggplot2" (Wickham, 2009) with the following parameters: stat = "smooth", method = "loess", span = 2. Based on the observed luminescence curves, we used data from day zero to six to calculate growth rates for all cell lines. For this, log-transformed luminescence intensities were fitted to a linear regression model. The quality of the linear fit is given by R squared and the luminescence doubling time (LDT) was calculated from the slope of the linear fit. The 95% confidence intervals were calculated for the LDT, intercept and AUC. The linear regression analysis was performed by the lm function in R, adjusted R squared values were calculated using R base function lm() with Wherry's equation (Team, 2015) and the AUC was calculated with the grofit package (Kahm et al., 2010).

MS-based analysis of proteome changes

For SILAC-based quantification of proteins (Ong et al., 2002), wild-type cells were grown in medium containing natural variants of L-arginine and L-lysine (Arg^0/Lys^0) and knockout cell lines in medium containing heavy isotope labeled variants of L-arginine and L-lysine (Arg^{10}/Lys^8). Cells were lysed in RIPA buffer (50 mM Tris-HCl pH 7.5, 150 mM NaCl, 1% Nonidet P-40, 0.1% sodium-dodecyl-sulfate, 1 mM EDTA) supplemented with protease inhibitors (Complete protease inhibitor mixture tablets, Roche Diagnostics). Lysates were incubated for 10 min on ice and cleared by centrifugation at 16,000 × g. An equal amount of protein from the "light" (wild-type) and the "heavy" (respective knockout cells) SILAC condition was mixed and SDS sample buffer added. Samples in SDS sample buffer were incubated with dithiothreitol (10 mM) for 10 min at 70 °C and alkylated with chloroacetamide (5.5 mM) for 60 min at 25 °C. Proteins were separated by SDS-PAGE using a 4%-12% gradient gel, visualized with colloidal coomassie blue stain, and digested using in-gel digestion method (Shevchenko et al., 2006). Gel lanes were cut into ten separate fractions and each gel piece was further sliced into small pieces (~1mm). Gel pieces were destained with 50% ethanol in 25 mM ammonium bicarbonate (pH 8.0) and dehydrated with 100% ethanol. Trypsin (in 25 mM ammonium bicarbonate pH 8.0) was added and incubated overnight. The trypsin digestion was stopped by addition of trifluoroacetic acid (0.5% final concentration) and peptides were eluted from the gel pieces by stepwise increase in acetonitrile concentration (to 100% final). Subsequently, acetonitrile was removed by centrifugal evaporation and peptides were purified by C18 reversed-phase packed Stage-Tips (Rappsilber et al., 2007). In a complementary approach cells were lysed in denaturing buffer (6 M urea, 2 M thiourea, 10 mM HEPES pH 8.0) and equal amounts of protein from "light" and "heavy" SILAC states were mixed. Protein were reduced with dithiothreitol (1 mM), alkylated with chloroacetamide (5.5 mM), four-fold-diluted with water and digested with trypsin overnight. The protein digestion was stopped by addition of trifluoroacetic acid (0.5% final concentration) and centrifuged at 16,000 × g for 10 min. Cleared peptides were fractionated by micro-column-based strong-cation exchange chromatography (SCX) into six fractions and cleaned by C18 reversed-phase packed Stage-Tips.

Peptide fractions were analyzed on a quadrupole Orbitrap (Q-Exactive, Thermo Scientific) mass spectrometer equipped with a nanoflow HPLC system (Thermo Scientific). Peptide samples were loaded onto C18 reversed-phase columns and eluted with a linear gradient from 8 to 40% acetonitrile containing 0.5% acetic acid in 2-3 hours. The Q-Exactive was operated in the data-dependent mode automatically switching between MS and MS-MS. Survey full-scan MS spectra (m/z 300–1700) were acquired in the Orbitrap. The 10 most intense ions were sequentially isolated

and fragmented by higher-energy C-trap dissociation (HCD). Peptides with unassigned charge states, as well as peptides with charge state less than +2 were excluded from fragmentation. Fragment spectra were acquired in the Orbitrap mass analyzer.

Raw MS data were analyzed by the MaxQuant software (version 1.5.0.38) (Cox and Mann, 2008). Mass spectra were searched against protein sequences from the UniProt knowledge base (downloaded January 2014) using the Andromeda search engine (Cox et al., 2011). Spectra were searched with a mass tolerance of 6 ppm for precursor ions, 20 ppm for fragment ions, strict trypsin specificity and allowing up to two missed cleavage sites. Cysteine carbamido methylation was searched as a fixed modification, whereas amino-terminal protein acetylation and methionine oxidation were searched as variable modifications. A false discovery rate of less than one percent was achieved using target-decoy search strategy (Elias and Gygi, 2007) and a posterior error probability filter. The raw data and MaxQuant output files have been deposited to the ProteomeXchange Consortium (http://proteomecentral.proteomexchange.org) via the PRIDE partner repository (Vizcaino et al., 2016) with the dataset identifier PXD003443.

Cell cycle profile analysis

Equal amounts of cells were seeded into three 6 cm dishes (20,000 cells per dish) and cultured for 12 hours before the start of the experiment. The experiment was performed by culturing cells in three different conditions- untreated, 48 hours reversine, and 96 hours reversine. The untreated cells were cultured during the entire experiment in medium without reversine and medium was changed after 48 hours. For the 48 hours reversine sample, cells were cultured in medium without reversine for the first 48 hours, and for the last 48 hours with medium containing reversine (0.5 μM). For the 96 hours reversine sample, cells were cultured during the entire experiment in medium with reversine (0.5 μM) and medium was changed after 48 hours. Cells from the three conditions (untreated, 48 hours reversine, 96 hours reversine) were trypsinized, washed twice in cold PBS and resuspended in 500 μl ice-cold PBS. Cells were permeablized by addition of 500 μl ice-cold ethanol, incubated for 45 min on ice, pelleted by centrifugation and washed with cold PBS. The cell pellet was resuspended in propidium iodide (PI) staining solution (10 μg/ml PI in PBS) supplemented with RNAse (25 μg/ml) and incubated for 30 min at 37 °C. Samples were analyzed with flow cytometry using a BD FACSCalibur (BD Biosciences), and the data were analyzed and visualized using the FlowJo software. To determine cell populations with different DNA content

(i.e. 2N, 2N-4N (including 4N), and >4N cell populations) gates were manually set for the wildtype untreated sample and applied to all samples of the same experiment.

Supplemental references

Cox, J., and Mann, M. (2008). MaxQuant enables high peptide identification rates, individualized p.p.b.-range mass accuracies and proteome-wide protein quantification. Nature biotechnology *26*, 1367-1372.

Cox, J., Neuhauser, N., Michalski, A., Scheltema, R.A., Olsen, J.V., and Mann, M. (2011). Andromeda: a peptide search engine integrated into the MaxQuant environment. Journal of proteome research *10*, 1794-1805.

Dynek, J.N., Goncharov, T., Dueber, E.C., Fedorova, A.V., Izrael-Tomasevic, A., Phu, L., Helgason, E., Fairbrother, W.J., Deshayes, K., Kirkpatrick, D.S., *et al.* (2010). c-IAP1 and UbcH5 promote K11-linked polyubiquitination of RIP1 in TNF signalling. Embo J *29*, 4198-4209.

Elias, J.E., and Gygi, S.P. (2007). Target-decoy search strategy for increased confidence in large-scale protein identifications by mass spectrometry. Nature methods *4*, 207-214.

Kahm, M., Hasenbrink, G., Lichtenberg-Frate, H., Ludwig, J., and Kschischo, M. (2010). grofit: Fitting Biological Growth Curves with R. Journal of Statistical Software. Journal of Statistical Software *33*, 1-21.

Ong, S.E., Blagoev, B., Kratchmarova, I., Kristensen, D.B., Steen, H., Pandey, A., and Mann, M. (2002). Stable isotope labeling by amino acids in cell culture, SILAC, as a simple and accurate approach to expression proteomics. Mol Cell Proteomics *1*, 376-386.

Rappsilber, J., Mann, M., and Ishihama, Y. (2007). Protocol for micro-purification, enrichment, pre-fractionation and storage of peptides for proteomics using StageTips. Nature protocols *2*, 1896-1906.

Shevchenko, A., Tomas, H., Havlis, J., Olsen, J.V., and Mann, M. (2006). In-gel digestion for mass spectrometric characterization of proteins and proteomes. Nature protocols *1*, 2856-2860.

Team, R.C. (2015). A language and environment for statistical computing.

Vizcaino, J.A., Csordas, A., Del-Toro, N., Dianes, J.A., Griss, J., Lavidas, I., Mayer, G., Perez-Riverol, Y., Reisinger, F., Ternent, T., *et al.* (2016). 2016 update of the PRIDE database and its related tools. Nucleic acids research *44*, D447-456.

Wickham, H. (2009). ggplot2: elegant graphics for data analysis (Springer New York).
