# Supplementary material for: The Spindle Assembly Checkpoint Is Not Essential for Viability of Human Cells with Genetically Lowered APC/C Activity
Source: Cell Rep. 2016 Feb 18;14(8):1829–40. doi: 10.1016/j.celrep.2016.01.060 (PMC4785794; doi:10.1016/j.celrep.2016.01.060)
Supplement: Document S2. Article plus Supplemental Information [file mmc3.pdf]

Cell Reports

Report

# The Spindle Assembly Checkpoint Is Not Essential for Viability of Human Cells with Genetically Lowered APC/C Activity

## Graphical Abstract

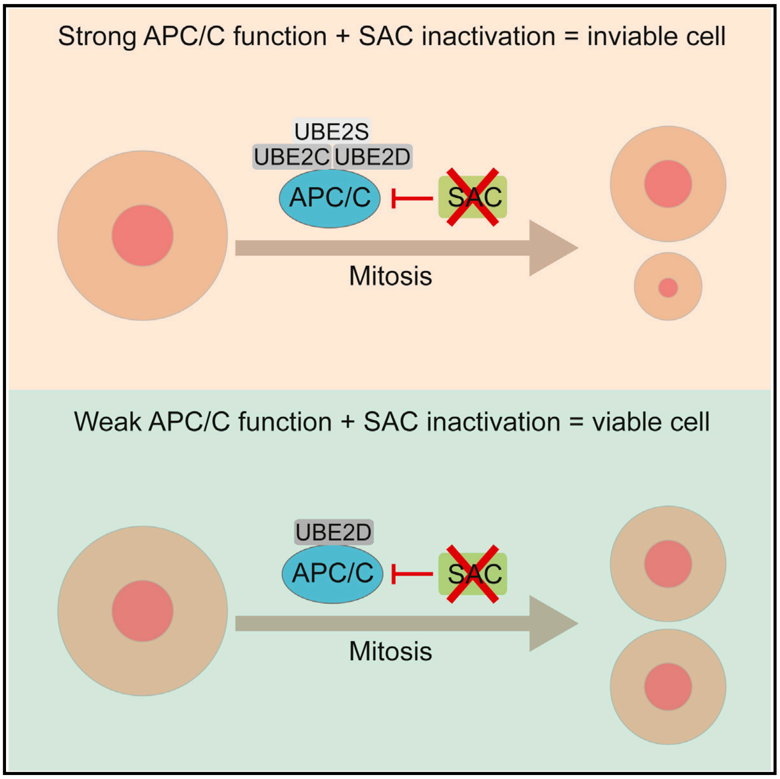


## Authors

Thomas Wild, Marie Sofie Yoo Larsen, Takeo Narita, Julie Schou, Jakob Nilsson, Chunaram Choudhary

## Correspondence

jakob.nilsson@cpr.ku.dk (J.N.),
chuna.choudhary@cpr.ku.dk (C.C.)

## In Brief

Wild et al. show that in mitosis the APC/C is activated by three E2 enzymes, which together elevate the APC/C to its maximum activity. Using chemical and genetic approaches, they show that it is the strength of the APC/C that makes the spindle assembly checkpoint essential in human cells.

## Highlights

- APC/C activity is powered by three E2 enzymes, UBE2S, UBE2C, and UBE2D
- UBE2S-catalyzed ubiquitylation has an important function in cells lacking UBE2C
- Reduction in APC/C activity renders the SAC unessential in human cells
- UBE2C-UBE2S deletion provides synthetic viability to MAD2 deletion

CrossMark

Wild et al., 2016, Cell Reports *14*, 1829–1840
March 1, 2016 ©2016 The Authors
http://dx.doi.org/10.1016/j.celrep.2016.01.060

CellPress

Cell Reports
Report

OPEN ACCESS
CellPress

# The Spindle Assembly Checkpoint Is Not Essential for Viability of Human Cells with Genetically Lowered APC/C Activity

Thomas Wild,[1,3] Marie Sofie Yoo Larsen,[2,3] Takeo Narita,[1] Julie Schou,[2] Jakob Nilsson,[2,*] and Chunaram Choudhary[1,*]
[1]Proteomics Program, the Novo Nordisk Foundation Center for Protein Research, Faculty of Health and Medical Sciences, University of Copenhagen, Blegdamsvej 3B, 2200 Copenhagen, Denmark
[2]Protein Signaling Program, the Novo Nordisk Foundation Center for Protein Research, Faculty of Health and Medical Sciences, University of Copenhagen, Blegdamsvej 3B, 2200 Copenhagen, Denmark
[3]Co-first author
*Correspondence: jakob.nilsson@cpr.ku.dk (J.N.), chuna.choudhary@cpr.ku.dk (C.C.)
http://dx.doi.org/10.1016/j.celrep.2016.01.060
This is an open access article under the CC BY license (http://creativecommons.org/licenses/by/4.0/).

## SUMMARY

**The anaphase-promoting complex/cyclosome (APC/C) and the spindle assembly checkpoint (SAC), which inhibits the APC/C, are essential determinants of mitotic timing and faithful division of genetic material. Activation of the APC/C is known to depend on two APC/C-interacting E2 ubiquitin-conjugating enzymes—UBE2C and UBE2S. We show that APC/C activity in human cells is tuned by the combinatorial use of three E2s, namely UBE2C, UBE2S, and UBE2D. Genetic deletion of *UBE2C* and *UBE2S*, individually or in combination, leads to discriminative reduction in APC/C function and sensitizes cells to *UBE2D* depletion. Reduction of APC/C activity results in loss of switch-like metaphase-to-anaphase transition and, strikingly, renders cells insensitive to chemical inhibition of MPS1 and genetic ablation of *MAD2*, both of which are essential for the SAC. These results provide insights into the regulation of APC/C activity and demonstrate that the essentiality of the SAC is imposed by the strength of the APC/C.**

## INTRODUCTION

The anaphase-promoting complex/cyclosome (APC/C) is a multi-subunit E3 ubiquitin ligase that is essential for eukaryotic cell division (Peters, 2006; Pines, 2011; Primorac and Musacchio, 2013; Sivakumar and Gorbsky, 2015). In mitosis, the APC/C promotes ubiquitylation-mediated degradation of key mitotic regulators, such as securin and cyclin B1 (CCNB1), which is required for metaphase-to-anaphase transition and cell division. The APC/C employs two E2 ubiquitin-conjugating enzymes, UBE2C (UBCH10) and UBE2S, in tandem, whereby UBE2C initiates substrate ubiquitylation and UBE2S subsequently extends substrate-linked ubiquitin to exclusively generate K11-linked polyubiquitin chains (Garnett et al., 2009; Summers et al., 2008; Williamson et al., 2009; Wu et al., 2010). In vitro, the APC/C also can initiate ubiquitylation using UBE2D (UBCH5) and subsequently extend it to polyubiquitin chains using UBE2S (Garnett et al., 2009; Williamson et al., 2009; Wu et al., 2010; Yu et al., 1996). However, the role of UBE2D in APC/C activation in vivo is unclear as depletion of UBE2D or expression of UBE2D mutants failed to reveal its function in mitosis (Bastians et al., 1999; Jin et al., 2008; Williamson et al., 2009).

The timing of APC/C activation in mitosis is tightly controlled by the spindle assembly checkpoint (SAC) (London and Biggins, 2014), which senses kinetochores that are not attached to microtubules and generates the mitotic checkpoint complex (MCC). The MCC inhibits APC/C activation until all chromosomes are properly aligned to the metaphase plate. Unattached kinetochores bind to monopolar spindle 1 (MPS1) kinase, which activates SAC signaling (Abrieu et al., 2001; Fisk and Winey, 2001; Hiruma et al., 2015; Ji et al., 2015; Santaguida et al., 2010). Activation of the SAC results in a conformational change of mitotic arrest-deficient protein 2 (MAD2 or MAD2L1) that is required for the formation of the MCC. Inactivation of the SAC in mammalian cells invariably leads to catastrophic aneuploidy, and, consequently, genetic deletions of SAC components are lethal (Kops et al., 2005).

Despite their central functions in mitosis, the functional redundancy of E2s in APC/C activity and the interdependence of the APC/C and the SAC are not fully understood. Here we used genetic approaches to delineate the function of APC/C-associated E2s and to assess the consequences of intermediate APC/C activity in cells. This enabled us to discover a UBE2C-independent role of UBE2S and to reveal a thus far concealed function of UBE2D in mitotic APC/C activity. Unexpectedly, we discovered that human cells with minimal APC/C activity, due to simultaneous deletion of UBE2C and UBE2S, lose their dependence on the SAC. We thus demonstrate that human cells, which normally rely on SAC activity for survival, can be engineered to be viable without the SAC. These results show that the essentiality of the SAC is imposed by the strength of the APC/C E2 module, and that human cells

CrossMark

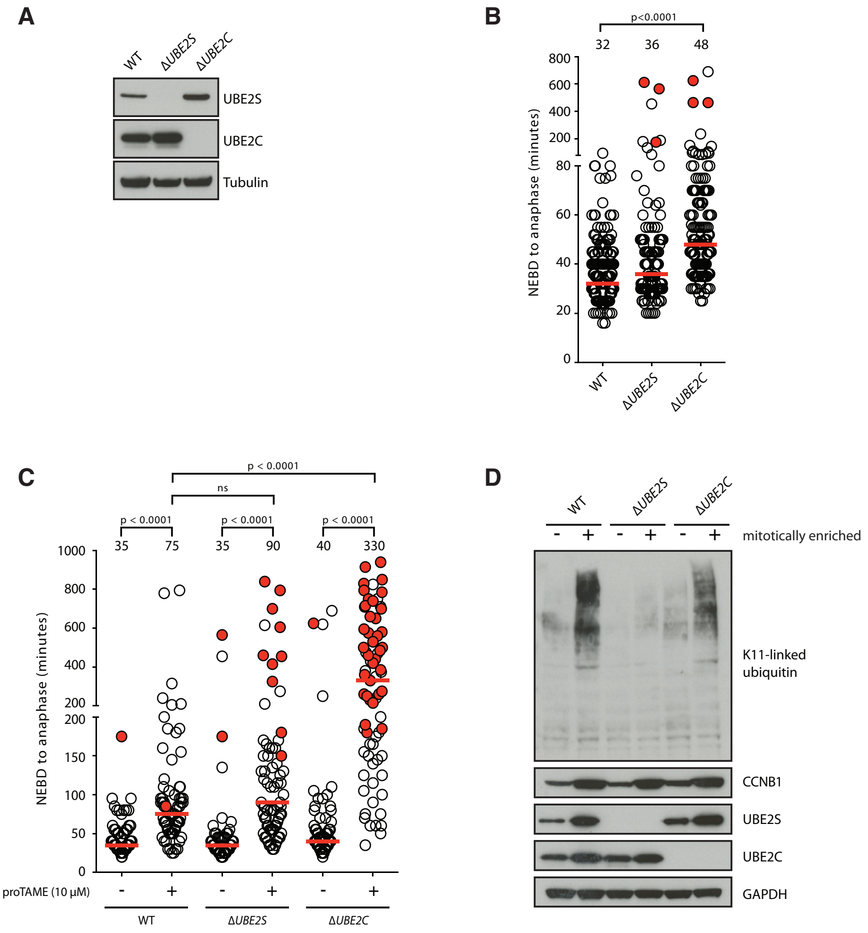


**Figure 1. Genetic Analysis of APC/C-Associated E2s Identifies UBE2C-Independent Function of UBE2S in Mitotic K11-Linked Ubiquitylation**

(A) Generation of *UBE2S*- and *UBE2C*-knockout HCT116 cells. Western blot analysis shows UBE2S and UBE2C levels in the indicated cell lines.

(B) NEBD-to-anaphase onset times in the indicated cells were measured by time-lapse differential interference contrast (DIC) microscopy. Each circle represents a single cell; open circles indicate cells that completed mitosis and red-filled circles indicate cells that did not exit mitosis within the stated time. The red line indicates median NEBD-to-anaphase onset time, which is noted on the top of the respective data points. For each cell line, at least 145 cells were analyzed from at least four independent experiments. The p values were calculated with Mann-Whitney tests.

(C) Cells were treated with the indicated concentrations of proTAME and NEBD-to-anaphase onset timing was measured by live-cell imaging (DIC), as in (B). At least 70 cells were analyzed from two independent experiments. The median is depicted as a red line and noted above the data points. The p values for the indicated conditions are stated on top (ns, $p \geq 0.01$).

(D) Analysis of K11-linked polyubiquitin chains in unsynchronized and mitotically enriched cells for the indicated cell lines. CCNB1 levels are shown to confirm enrichment of mitotic cells.

can acquire a genetic state in which the SAC becomes unessential.

## RESULTS

### UBE2C and UBE2S Are Dispensable for Mitosis and Cell Viability

To investigate the requirement of E2s for APC/C activity in mitosis, we generated *UBE2C*- and *UBE2S*-knockout (Δ*UBE2C* and Δ*UBE2S*) HCT116 (a human colorectal carcinoma cell line) cells using the clustered regularly interspaced short palindromic repeats (CRISPR)/Cas9 technology (Cong et al., 2013; Mali et al., 2013; Figure 1A). Function of the APC/C in these cells was assessed by quantifying the duration of nuclear envelope breakdown (NEBD) to anaphase onset (Figure 1B). Compared to wild-type (WT) cells, Δ*UBE2C* cells displayed a significant ($p < 0.0001$) delay in anaphase onset after NEBD, whereas ablation of *UBE2S* caused only a relatively minor delay. These results showed that UBE2S and UBE2C have a function in mitosis, but they are not essential for mitosis and cell viability. To confirm whether mitotic delay in Δ*UBE2C* cells resulted from impaired APC/C activity, we assayed the sensitivity of these cells to proTAME, a small molecule inhibitor of the APC/C (Zeng et al., 2010). Indeed, Δ*UBE2C* cells displayed a greater sensitivity to proTAME compared to WT and Δ*UBE2S* cells (Figure 1C), consistent with impaired APC/C activity in these cells. This result is also in agreement with the prolonged NEBD-to-anaphase onset timing in Δ*UBE2C* cells (Figure 1B).

### UBE2S Can Generate K11-Linked Polyubiquitin Chains in the Absence of UBE2C

Because UBE2S alone cannot initiate APC/C-mediated ubiquitylation (Garnett et al., 2009), it is conceivable that Δ*UBE2C* cells also lack UBE2S-dependent APC/C function, possibly explaining the more severe phenotypes seen in Δ*UBE2C* cells compared to Δ*UBE2S* cells. To test this hypothesis, we assessed the mitosis-specific increase in K11-linked ubiquitylation, which depends on UBE2S activity (Williamson et al., 2009). We observed a strong increase in K11 linkages in mitotically enriched WT cells, and, consistent with previous RNAi-based data (Matsumoto et al., 2010; Williamson et al., 2009), this increase was abrogated in Δ*UBE2S* cells (Figure 1D). While deletion of *UBE2C* reduced mitotic K11 ubiquitylation, a significant pool of K11-linked ubiquitin was still present in these cells, clearly demonstrating that in vivo UBE2S also can generate polyubiquitin chains independently of UBE2C.

### APC/C Activity Is Severely Impaired in *UBE2C* and *UBE2S* Double Knockouts

The inability of UBE2S to initiate APC/C-dependent ubiquitylation (Garnett et al., 2009; Williamson et al., 2009; Wu et al., 2010) suggested that the viability of *ΔUBE2C* cells (Figure 1A; Li et al., 2014) cannot be explained by the presence of UBE2S in these cells. Instead, the presence of K11-linked ubiquitylation in mitotically enriched *ΔUBE2C* cells, but not in *ΔUBE2S* cells, suggested that UBE2S extends ubiquitylation catalyzed by another E2 that cooperates with the APC/C to initiate substrate ubiquitylation. Therefore, we surmised that such an E2 may be sufficient to provide minimum APC/C function in the absence of UBE2C and UBE2S. Indeed, by deleting *UBE2C* in Δ*UBE2S* cells, we were able to obtain four clonal cell lines (#3, #4, #8, and #12) that were deficient for both APC/C-specific E2s (Figure 2A). NEBD-to-anaphase onset timing was severely prolonged in Δ*UBE2S*Δ*UBE2C* cell clones (Figure 2B). Thus, simultaneous deletion of *UBE2S* and *UBE2C* has an aggravated effect on mitotic progression compared to deletion of either gene individually. This result further points to UBE2S function that is independent of UBE2C, consistent with the notable increase in mitotic K11 linkages in *ΔUBE2C* cells (Figure 1D). The APC/C is essential for mitosis and it is, therefore, unlikely that Δ*UBE2S*-Δ*UBE2C* entirely lacked APC/C function. To formally test the APC/C activity in the absence of UBE2S and UBE2C, we treated Δ*UBE2S*Δ*UBE2C* cells with proTAME. Compared to WT cells, Δ*UBE2S*Δ*UBE2C* cells displayed a markedly increased sensitivity to proTAME (Figure 2C), providing evidence for the activity of the APC/C in these cells and demonstrating that the APC/C can function without these two E2s.

### UBE2D Functions with the APC/C In Vivo

The above results clearly indicate a role of another (separate from UBE2C and UBE2S) E2 enzyme in APC/C function. In vitro, UBE2D can support APC/C-dependent substrate ubiquitylation, and UBE2S can promote subsequent polyubiquitylation of these substrates (Garnett et al., 2009). Thus, UBE2D is an attractive candidate that could mediate UBE2C- and UBE2S-independent APC/C activity, but previous studies have questioned its functional relevance in vivo (Bastians et al., 1999; Jin et al., 2008; Williamson et al., 2009). To test whether UBE2D mediates APC/C activity in Δ*UBE2S*Δ*UBE2C* cells, *UBE2D* was depleted in these cells using RNAi. The UBE2D family of E2s is among the most promiscuous and can function with a large number of E3 enzymes (Komander and Rape, 2012). Therefore, to minimize pleiotropic effects of strong *UBE2D* depletion, we established RNAi conditions resulting in a relatively modest knockdown (Figure S1A). While modest *UBE2D* depletion had no discernible effect on mitosis in WT cells, all tested Δ*UBE2S*Δ*UBE2C* cell clones displayed a significantly prolonged mitosis upon *UBE2D* knockdown (Figure 2D; Figure S1A). Notably, *UBE2D* knockdown also exacerbated the mitotic delay in Δ*UBE2C* cells, but not in Δ*UBE2S* cells (Figure 2E; Figure S1B). The most likely explanation for this observation is that UBE2S cannot function in the absence of UBE2C and UBE2D, which is consistent with biochemical data showing that UBE2S can extend ubiquitin linkages but cannot initiate substrate ubiquitylation. Together, these results show that UBE2C and UBE2D can provide sufficiently robust APC/C function in the absence of UBE2S and that they function independent of each other with the APC/C. While UBE2D alone can support minimal APC/C activity, its function is notably strengthened by the presence of UBE2S, most likely by extending UBE2D-dependent, APC/C-initiated ubiquitylation. Together, our results establish that three E2 enzymes, UBE2C, UBE2S, and UBE2D, function as bona fide partners for the APC/C in vivo.

### Δ*UBE2S*Δ*UBE2C* Cells Lose Switch-like Metaphase-to-Anaphase Transition

To better understand the basis for the delay in NEBD to anaphase onset in Δ*UBE2S*Δ*UBE2C* cells, we measured the duration of NEBD to metaphase plate formation and to subsequent anaphase onset (Figures S2A and S2B). Δ*UBE2S*Δ*UBE2C* cells required a longer time to establish the metaphase plate and showed delays in the metaphase-to-anaphase transition, indicating that APC/C function in these cells is severely impaired (Figure S2A). To more directly assess mitotic APC/C activity in the knockout cells, we analyzed the degradation kinetics of Venus-tagged endogenous CCNB1 (cyclin B1), a key substrate of the APC/C for mitotic exit (Irniger et al., 1995; King et al., 1995; Sudakin et al., 1995). In WT cells, as well as in Δ*UBE2S* and Δ*UBE2C* single-knockout cells, CCNB1 was degraded rapidly after the establishment of the metaphase plate (Figures 2F and 2G; Figures S3A and S3B), but CCNB1 degradation was significantly impaired in Δ*UBE2S*Δ*UBE2C* cells. These results show that reduced APC/C activity, due to simultaneous deletion of *UBE2S* and *UBE2C*, leads to loss of rapid onset of CCNB1 degradation upon establishment of the metaphase plate. Thus, the strength of the APC/C is important for switch-like metaphase-to-anaphase transition.

### Lowering APC/C Activity Renders the SAC Non-essential for Cell Viability

During mitosis, formation of the metaphase plate leads to SAC inactivation and triggers APC/C activation, which in turn prompts rapid anaphase onset. Therefore, to test SAC activity in Δ*UBE2S*Δ*UBE2C* cells, we treated cells with nocodazole, which inhibits microtubule polymerization and potently activates the SAC. As expected, nocodazole treatment caused severe mitotic arrest in WT as well as in Δ*UBE2C* and Δ*UBE2S* single-knockout cells (Figure 3A). Interestingly, one of the Δ*UBE2S*Δ*UBE2C* clones displayed a marked insensitivity to nocodazole, indicating a severely compromised SAC in this particular cell line. Because the SAC is indispensable for mammalian cells, severe insensitivity of Δ*UBE2S*Δ*UBE2C* #4 to nocodazole indicated that SAC may not be essential for viability of these cells. This prompted us to directly assess the requirement of SAC function for viability of all Δ*UBE2S*Δ*UBE2C* cell clones. We treated cells with reversine to inhibit the kinase MPS1 that is essential for initiating the SAC (Abrieu et al., 2001; Fisk and Winey, 2001; Santaguida et al., 2010). Inhibition of MPS1 results in improper chromosome segregation, which invariably results in cell death. Reversine potently inhibited proliferation of WT, Δ*UBE2S*, and Δ*UBE2C* cells (Figure 3B), confirming that MPS1 activity is essential for these cells. Strikingly, all Δ*UBE2S*Δ*UBE2C* cell clones, irrespective of their SAC activity

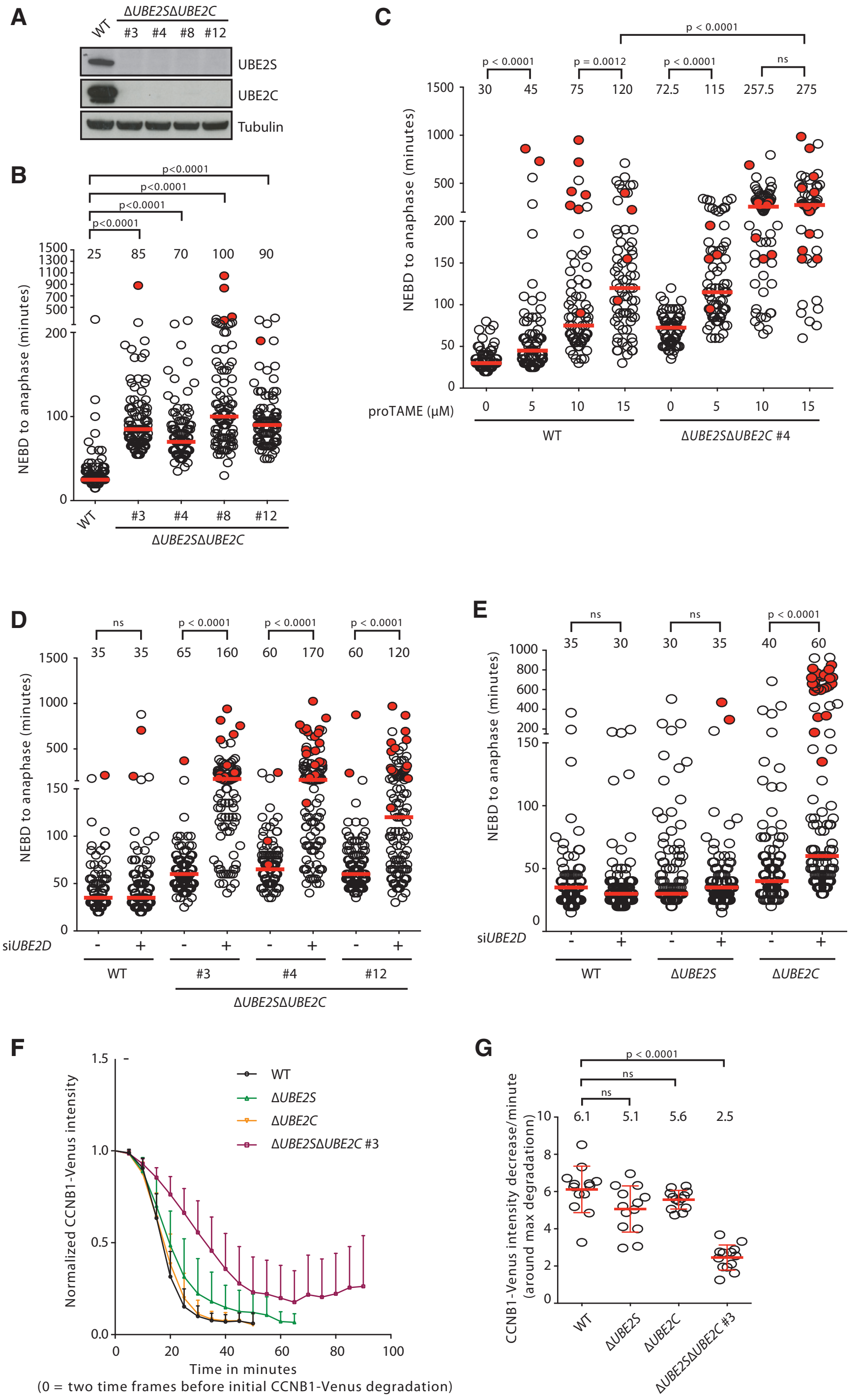


**Figure 2. Genetic Deletion of APC/C-Specific E2s Uncovers In Vivo Function of UBE2D in APC/C Activation**

(A) Four independent Δ*UBE2S*Δ*UBE2C* cell clones were generated, and deletion of UBE2S and UBE2C was confirmed by immunoblotting.

(B) NEBD-to-anaphase onset times in Δ*UBE2S*Δ*UBE2C* cells, analyzed as described in Figure 1B. For each cell line, at least 87 cells were analyzed from four independent experiments.

(C) Cells were treated with the indicated concentrations of proTAME and NEBD-to-anaphase onset timing was measured by live-cell imaging (DIC). At least 55 cells were analyzed from two independent experiments. The red line indicates the median NEBD-to-anaphase time, which is noted above the data points. The p values for the indicated conditions are stated on top (ns, $p \geq 0.01$).

(D) NEBD-to-anaphase onset timing was analyzed as described in Figure 1B. 24 hr prior to filming, WT and the indicated Δ*UBE2S*Δ*UBE2C* cell clones were treated with small interfering RNAs (siRNAs) targeting *UBE2D* (+) or control (–) siRNA. For each condition, at least 113 cells were analyzed from three independent experiments (ns, $p \geq 0.01$).

(E) Cells were treated with siRNAs targeting UBE2D (+) or control siRNA (–), and NEBD-to-anaphase onset timing was measured by live-cell imaging (DIC). At least 95 cells were analyzed from three independent experiments. The median is depicted as a red line and the median time is shown above the data points. The p values for the indicated conditions are stated on top (ns, $p \geq 0.01$).

(F) CCNB1 degradation curves for the indicated cell lines. One allele of CCNB1 was tagged with Venus, and degradation of CCNB1-Venus was analyzed by live-cell imaging. Time = 0 is set to two frames before the beginning of CCNB1 degradation, which was defined as a decrease in CCNB1-Venus intensity of >4%. The curves depict mean CCNB1-Venus fluorescent intensity from analyzed cells (from time = 0 to five time frames after anaphase onset), and the one-sided error bars show the SD.

(G) CCNB1 degradation rates were calculated from the experiment shown in (F). The best linear fit around the maximum difference in CCNB1-Venus intensities (normalized to NEBD) between two time points was determined, and the corresponding slopes are plotted. The mean values are noted on the top of the data points and depicted as a red line with SDs emerging as vertical lines. The p values were calculated with t tests (ns, $p \geq 0.01$).

See also Figures S1–S3.

in nocodazole experiments (Figure 3A), grew in the presence of reversine (Figure 3B; Figure S4A). The resistance of Δ*UBE2S*Δ*UBE2C* cells to reversine indicated that the SAC is not essential in Δ*UBE2S*Δ*UBE2C* cells.

To unequivocally test this, we tried to genetically delete *MAD2*, an essential gene and key component of the SAC (Michel et al., 2004), in Δ*UBE2S*Δ*UBE2C* cells. We used two independent Δ*UBE2S*Δ*UBE2C* cell clones (#3 and #4), which differed in their sensitivity to nocodazole and, thus, in SAC functionality (Figure 3A). Indeed, we succeeded in obtaining *MAD2*-deficient cells in the Δ*UBE2S*Δ*UBE2C* background (Figure 3C; Figure S4B), clearly demonstrating that the SAC function is no longer essential after *UBE2C* and *UBE2S* deletion. As expected, in WT cells, RNAi against *MAD2* resulted in aberrant mitosis, as

marked by accelerated mitotic timing and failure to form a proper metaphase plate (Figure 3D; Figures S4C–S4F). Notably, Δ*UBE2S*Δ*UBE2C*Δ*MAD2* cells displayed normal metaphase plate formation and segregation of chromosomes despite complete lack of MAD2. Thus, the mitotic phenotype of Δ*UBE2S*Δ*UBE2C*Δ*MAD2* cells is dissimilar from WT cells depleted of *MAD2* by RNAi and, rather, they resemble control WT cells (Figure 3D). Genetic deletion of SAC components results in grossly abnormal mitosis, and these mitosis-incompetent cells eventually are eliminated through cell death. Thus, viability of *ΔUBE2SΔUBE2CΔMAD2* cells, without gross abnormalities in chromosomal separation, is striking. We speculate that prolonged mitotic timing in *ΔUBE2SΔUBE2CΔMAD2* cells, compared to WT cells (Figure S4C), may be sufficient to allow for proper chromosome alignment in the absence of a functional SAC. Consistent with this notion, inhibition of the SAC by reversine in WT, Δ*UBE2S*, and Δ*UBE2C* cells caused massive polyploidy (Figure 3E). In stark contrast, reversine had no observable effect on the ploidy of Δ*UBE2S*Δ*UBE2C* and Δ*UBE2S*Δ*UBE2C*Δ*MAD2* cells (Figure 3E; Figure S4G). These results establish that the SAC activity becomes dispensable for proliferation in human cells lacking both *UBE2S* and *UBE2C*. Thus, reducing APC/C activity confers synthetic viability to MPS1 inhibition and *MAD2* deletion.

## Distinct Consequences of SAC Inhibition in Δ*UBE2S*Δ*UBE2C* and *TP53*-Knockout Cells

A previous study reported viability of murine embryonic fibroblasts (MEFs) with simultaneous deletion of *TP53* and *MAD2* (Burds et al., 2005). However, unlike our Δ*UBE2S*Δ*UBE2C*Δ*MAD2* HCT116 cells, the reported Δ*TP53*Δ*MAD2* MEFs showed extreme chromosome instability (with >50% of mitotic cells showing chromosome missegregation) and aneuploidy. To directly compare the consequence of *TP53* deletion and the APC/C weakening upon SAC inactivation in human cells, we used HCT116 *TP53*-knockout cells (Bunz et al., 1998) and inactivated the SAC by two different approaches as follows: (1) inhibiting the kinase MPS1 with reversine, and (2) knocking down *MAD2* using RNAi. In contrast to Δ*UBE2S*Δ*UBE2C* cells, proliferation of *TP53*-knockout cells was inhibited by reversine, albeit they showed improved viability compared to WT cells (Figure 4A). Also, reversine-treated WT and *TP53*-knockout cells were multinucleated and grossly polyploid; in sharp contrast, the ploidy of Δ*UBE2S*Δ*UBE2C* cells was not affected by reversine treatment (Figures 4B and 3E). Ablation of MAD2 accelerated mitosis in WT, Δ*UBE2S*Δ*UBE2C*, and *TP53*-knockout cells (Figure 4C). Notably, the mitotic duration of *MAD2*-depleted Δ*UBE2S*Δ*UBE2C* cells, but not *MAD2*-depleted *TP53*-knockout cells, was comparable to the mitotic duration of untreated WT cells (i.e., not depleted of *MAD2*). Consistent with these results, *MAD2* depletion in *TP53*-knockout cells resulted in severe problems in chromosome segregation compared to Δ*UBE2S*Δ*UBE2C* cells (Figure 4D). Thus, our results demonstrate that in human cells deletion of *TP53* is not sufficient to prevent chromosome segregation problems caused by SAC inactivation. These results show that the consequences of *TP53* deletion and lowering the APC/C activity on the requirement of the SAC are fundamentally distinct.

## Weak SAC Activity Can Be Beneficial in Cells with Compromised APC/C Function

To explore how Δ*UBE2S*Δ*UBE2C* #4 reduced SAC activity (Figure 3A), we quantified changes in protein expression using stable isotope labeling by amino acids in cell culture (SILAC)-based proteomics (Table S1). Notably, we found that levels of MPS1 were reduced in this clone, and this observation subsequently was confirmed independently by western blotting (Figure 5A). Because MPS1 is essential for activation of the SAC, we reasoned that the downregulation of MPS1 specifically in this clone could have led to reduced SAC activity. Consistently, ectopic expression of TFP-MPS1 in these cells restored SAC activity (Figure 5B). The SAC-generated MCC is a direct inhibitor of the APC/C; thus, it is an attractive possibility that dampening of SAC activity, for example, by downregulating MPS1, could actually be beneficial in the context of low APC/C activity.

To test this hypothesis, we wished to generate new Δ*UBE2S*Δ*UBE2C* cells in which SAC activity could be restored at will. To do this, we deleted *UBE2C* in Δ*UBE2S* cells in the presence of reversine, thereby chemically mimicking the low MPS1 levels observed in the Δ*UBE2S*Δ*UBE2C* #4 (Figure 5C). Using this approach, we obtained double-knockout cell clones (termed Δ*UBE2S*Δ*UBE2C*-R), and, unless indicated, these cells were cultured continuously in reversine (Figure 5D). To test if SAC inactivation in these cells is inconsequential or beneficial, we restored SAC function by removing reversine and assessed their proliferation. Indeed, Δ*UBE2S*Δ*UBE2C*-R cells grew better in the presence of reversine than in its absence (Figure 5E). It should be noted that reversine treatment resulted in a reduction in proliferation of both Δ*UBE2S*Δ*UBE2C* and Δ*UBE2S*Δ*UBE2C*Δ*MAD2* cells (Figure 3B; Figures S5A and S5B), indicating a SAC inhibition-independent adverse effect of reversine on cell proliferation. Thus, we interpret that, in Δ*UBE2S*Δ*UBE2C*-R cells, the benefit of reversine-mediated SAC inhibition outweighs the SAC-independent growth disadvantage caused by reversine. In agreement with this, removal of reversine in Δ*UBE2S*Δ*UBE2C*-R cells led to increased timing from NEDB to anaphase onset (Figure 5F), as NEDB-to-anaphase onset timing was almost doubled 3 days after removing reversine. Importantly, the increase in mitotic timing after reversine removal was SAC dependent, as depletion of *MAD2* by RNAi substantially ameliorated this defect (Figure 5G). Hence, low SAC activity can be of avail for cells with critically low APC/C activity.

## DISCUSSION

In this work we report several findings: (1) we show that UBE2S can catalyze mitotic K11 ubiquitin linkages in vivo in the complete absence of UBE2C and that the deletion of UBE2S in UBE2C background causes a more pronounced cellular phenotype than deletion of UBE2C alone, thus revealing an important function of UBE2S that is independent of UBE2C. (2) We show that UBE2C-UBE2S double-knockout cells are viable. While mitosis in these cells was severely prolonged, their viability demonstrates that the APC/C can function without its known physiological E2 module (Jin et al., 2008; Williamson et al., 2009; Wickliffe et al., 2011). (3) We provide in vivo evidence that UBE2D functions with the APC/C in mitosis, which is consistent

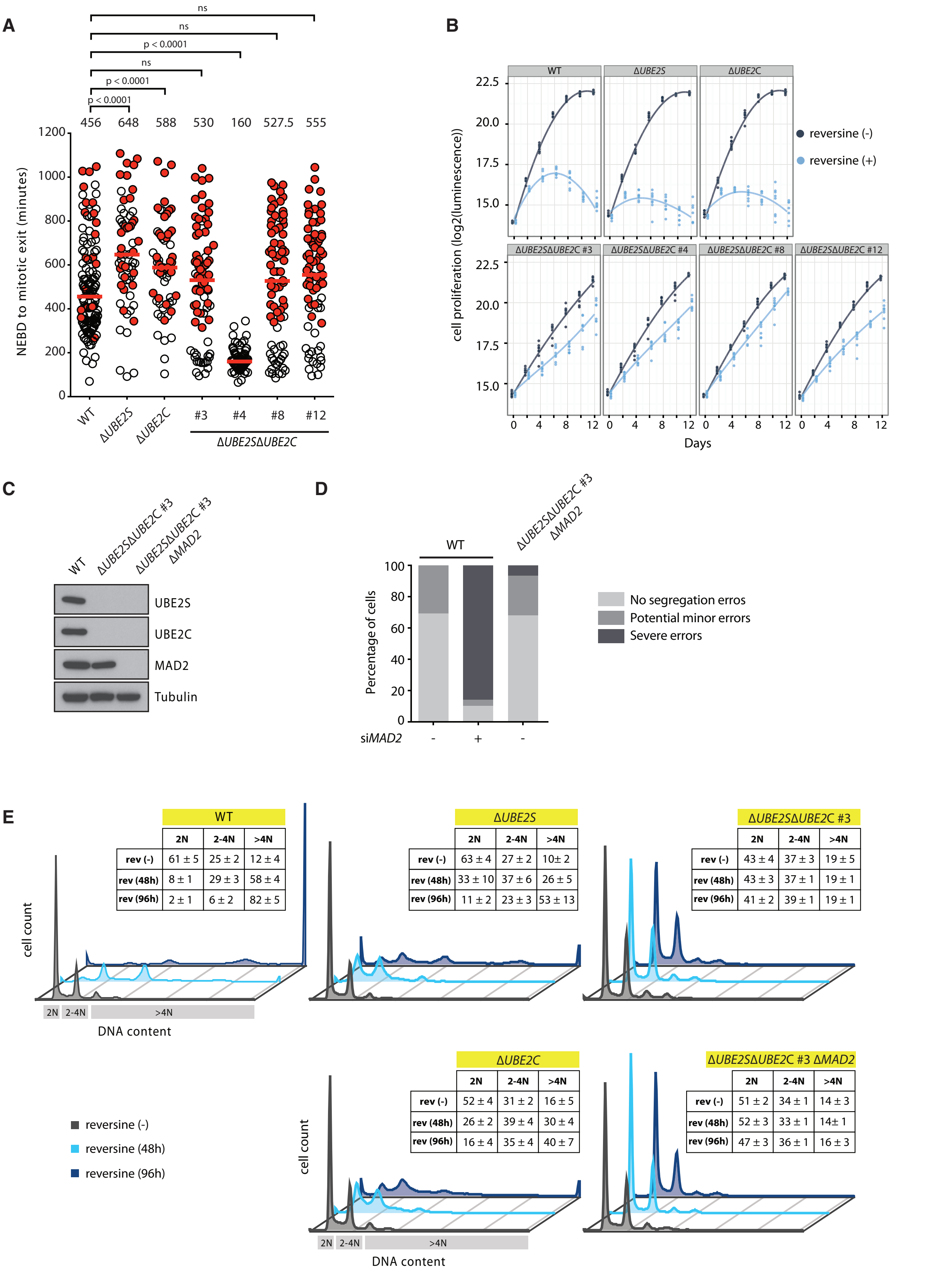


(legend on next page)

with previous in vitro data (Garnett et al., 2009; Summers et al., 2008; Williamson et al., 2009; Wu et al., 2010) and a recently reported function of UBE2D3 in meiosis of murine oocytes (Ben-Eliezer et al., 2015). (4) We show that a reduction in APC/C function provides synthetic viability to SAC inactivation, as cells lacking MAD2 and MPS1 functions are viable in the context of low APC/C activity.

Our results establish that in human cells the activity of the APC/C is powered by three different E2s: UBE2C, UBE2S, and UBE2D. Deletion of UBE2S, which catalyzes mitosis-specific K11-linked ubiquitylation, only modestly affected mitotic timing. These results are consistent with previous RNAi-based studies (Meyer and Rape, 2014; Williamson et al., 2009), and they clearly show that weak mitotic phenotypes observed in previous studies are not due to incomplete knockdown of UBE2S. These results show that UBE2S, and thus UBE2S-catalyzed mitotic upregulation of K11 linkages, is not absolutely essential for mitotic progression. Deletion of APC/C-associated E2s, individually and in combination, allowed us to establish an important function of UBE2S in mitosis, as we demonstrate that Δ*UBE2S*Δ*UBE2C* cells have significantly prolonged mitosis timing compared to Δ*UBE2C* cells. Previous studies have shown that RNAi-mediated depletion of *UBE2C* together with *UBE2S* stabilizes APC/C substrates and delays mitosis more efficiently than either single depletion (Williamson et al., 2009). However, these results were interpreted as evidence for UBE2C and UBE2S constituting a functional pair for APC/C-mediated ubiquitylation and not as evidence for an UBE2C-independent function of UBE2S. In this work we reveal an UBE2C-independent function of UBE2S. Our results are consistent with the emerging notion that UBE2S-catalyzed polyubiquitylation of APC/C substrates is not absolutely necessary for their degradation but can increase the rate of substrate degradation (Dimova et al., 2012; Garnett et al., 2009; Meyer and Rape, 2014).

While UBE2C and UBE2S are APC/C-specific E2 enzymes, the UBE2D family of E2s functions in conjunction with many (possibly hundreds of) E3 enzymes and has been implicated in many other cellular processes (Komander and Rape, 2012). Thus, it is difficult to genetically assess the relative contribution of UBE2D to APC/C function in vivo. Regardless, we show that Δ*UBE2S*Δ*UBE2C* cells display a pronounced mitotic delay and reduced CCNB1 degradation compared to Δ*UBE2C* cells, clearly indicating that the APC/C can initiate its substrate ubiquitylation independent of UBE2C. Furthermore, we show that knockdown of *UBE2D* in Δ*UBE2C* cells exacerbates mitotic delay and that UBE2S can catalyze K11-linked polyubiquitylation in mitotic Δ*UBE2C* cells, likely by extending UBE2D-APC/C-initiated ubiquitylation. These findings, and previous reports that UBE2D can promote APC/C-dependent ubiquitylation in vitro (Garnett et al., 2009; Williamson et al., 2009; Wu et al., 2010; Yu et al., 1996), support the functional relevance of UBE2D in mediating APC/C activity in vivo. Importantly, we did not observe increased UBE2D levels upon deletion of UBE2C (Figure S1; Table S1), indicating that normal cellular UBE2D levels are sufficient to result in notable UBE2D-dependent APC/C activity. We speculate that UBE2D could provide basal activity to the APC/C, and mitosis-specific upregulation of UBE2C and UBE2S leverages the APC/C to its maximum power. Thus, the combinatorial use of the three different E2s confers robustness and tunability to mitotic APC/C activity. This also may explain why we, and other researchers (Bastians et al., 1999; Jin et al., 2008; Williamson et al., 2009), have failed to note a clear mitotic phenotype upon UBE2D depletion in WT cells, as the other two E2s would maintain sufficient APC/C activity upon UBE2D depletion.

Importantly, this work shows that it is the strength of the APC/C, powered by three cooperating E2s, that renders the SAC essential in human cells. In a simple model, the APC/C and the SAC can be viewed as mitotic accelerator and brake, respectively. While the accelerator is strictly required to progress through mitosis, the brake is only needed in proportion to the strength of the accelerator and a strong brake could even be detrimental to cells with a weak accelerator. Notably, the SAC is essential in all metazoans, with the exception of *Drosophila* (Buffin et al., 2007). To the best of our knowledge, we report the first example of long-term viable human cells lacking the SAC. Lowering APC/C activity renders the SAC non-essential in human cells, which normally rely on SAC activity for viability, possibly by extending the available time for aligning the chromosomes. Interestingly, genetic screens in *C. elegans* identified

**Figure 3. UBE2S and UBE2C Jointly Render the SAC Essential for Cell Viability**

(A) NEBD-to-anaphase onset timing for the indicated cell lines upon nocodazole treatment (30 ng/ml) measured by time-lapse microscopy (DIC). Each circle represents a single cell (red-filled circles indicate cells that did not exit mitosis within the stated time). At least 61 cells were analyzed from two independent experiments. The median is depicted as a red line and noted above the data points. The p values for the indicated comparisons were calculated with Mann-Whitney tests (ns, $p \geq 0.01$).

(B) Growth analysis of the indicated cell lines in the presence or absence of 0.5 μM reversine. Cell proliferation was assessed at the indicated times using a luminescence-based assay measuring ATP levels. A local regression curve is plotted for each cell line and condition based on at least six measurements per time point.

(C) Generation of *UBE2S*, *UBE2C*, and *MAD2* triple-knockout cells. The immunoblot confirms deletion of the indicated proteins in the parental Δ*UBE2S*Δ*UBE2C* #3 and the triple-knockout clone.

(D) Comparison of mitotic phonotypes of *MAD2*-deleted Δ*UBE2S*Δ*UBE2C* #3 cells with WT cells in which *MAD2* was knocked down with RNAi. Cells transiently transfected with RFP-tagged histone H2B were monitored by live-cell imaging and classified into categories representing normal and aberrant mitotic phenotypes. At least 27 cells were analyzed from three independent experiments.

(E) The histograms depict DNA content (2N, 2–4N, and >4N; N approximates a haploid genome) in the specified cells that were cultured with or without reversine (0.5 μM) for the indicated time (hr). The inlays show percentages of cells with the indicated DNA content for the specified cells and conditions; the values show mean ± SDs from three independent experiments. DNA content was analyzed by propidium iodide staining and flow cytometric analysis. For each experiment, a minimum of 9,000 cells were analyzed for each condition. The histograms show representative data from one of the experiments. rev, reversine.

See also Figure S4.

OPEN
ACCESS
CellPress

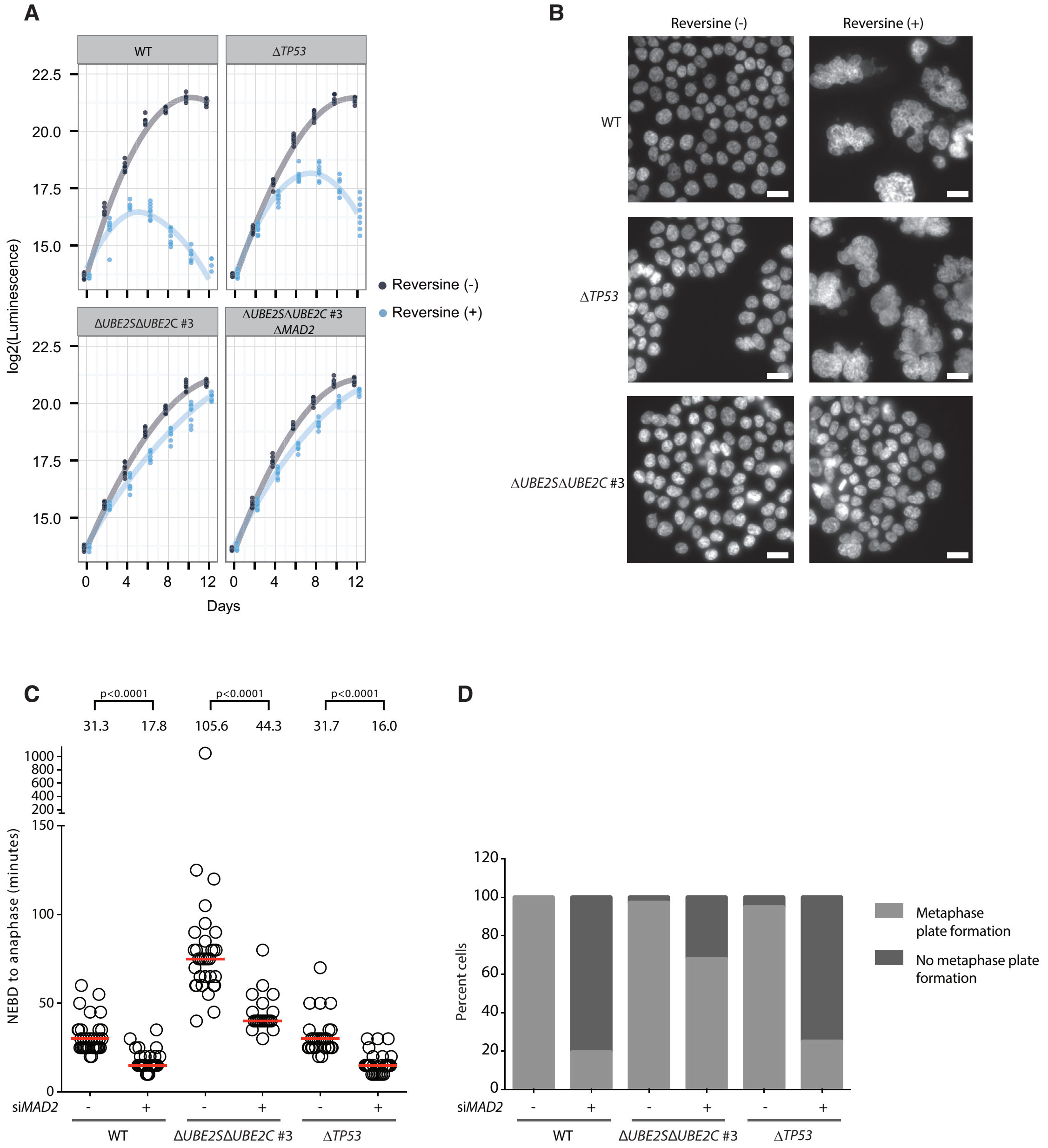


**Figure 4. Analysis of SAC Inhibition in Δ*UBE2S*Δ*UBE2C* and *TP53*-Knockout Cells**

(A) Growth analysis of Δ*UBE2S*Δ*UBE2C* cells and *TP53*-knockout cells in the presence or absence of 0.5 μM reversine. Cell proliferation was assessed at the indicated times using a luminescence-based assay measuring ATP levels. A local regression curve is plotted for each cell line and condition based on at least six measurements per time point.

(B) Inhibition of MPS1 causes polyploidy in HCT116 WT and Δ*TP53* cells, but not in Δ*UBE2S*Δ*UBE2C* cells. Cells were treated with reversine for 4 days, and medium was changed after 2 days in culture. Cellular DNA was stained with Hoechst and imaged using a wide-field microscope. The scale bar represents 20 μm.

(C) Knockdown of *MAD2* accelerates mitotic progression. Cells transiently transfected with *MAD2* siRNAs and RFP-tagged histone H2B were monitored by time-lapse microscopy. NEBD to anaphase onset for the indicated cell lines was analyzed; each circle represents a single cell. The p values were calculated with Mann-Whitney tests.

(D) Comparison of mitosis of WT, Δ*UBE2S*Δ*UBE2C*, and Δ*TP53* cells upon *MAD2* knockdown. Formation of the metaphase plate in mitotic cells was analyzed from the experiment shown in (C). Note that knockdown of *MAD2* in WT and Δ*TP53* cells leads to gross problems in metaphase plate formation.

temperature-sensitive mutations in APC/C subunits and its mitotic co-activator CDC20 as suppressors of the lethality caused by loss of the essential SAC component MAD1 (Furuta et al., 2000; Kitagawa et al., 2002; Tarailo et al., 2007). These studies established that in *C. elegans* a mitotic delay can compensate for the lack of the normally essential SAC. In line

with this notion, our results demonstrate that in human cells the only essential function of the SAC is to restrain the APC/C activity in mitosis in order to provide sufficient time for aligning the chromosomes to the metaphase plate.

A previous study (Burds et al., 2005) showed that simultaneous deletion of *TP53* and *MAD2* led to very high rate of chromosome instability. Importantly, our engineered (Δ*UBE2S*Δ*UBE2C*) cells are viable without the SAC in the presence of p53. We demonstrate that the consequences of *TP53* inactivation and the reduction in the APC/C activity upon inactivation of SAC function are fundamentally different. The key distinction is that deletion of *UBE2C* and *UBE2S* prevents SAC inactivation-triggered aneuploidy, whereas deletion of *TP53* does not. Without the SAC, deletion of *TP53* can apparently dampen the consequences of a catastrophic mitosis (i.e., polyploidy-driven apoptosis), whereas weakening of the APC/C (by means of *UBE2C* and *UBE2S* deletion) prevents this catastrophic mitosis in the first place.

Small molecule inhibitors of MPS1 and the APC/C are being explored for therapeutic targeting of cancers (Liu and Winey, 2012; Sackton et al., 2014; Zeng et al., 2010). Here we discovered a genotype, Δ*UBE2S*Δ*UBE2C*, that allows cells to tolerate chemical inhibition of MPS1, providing possible means of resistance. Inversely, incomplete APC/C inhibition could be compensated by reduced SAC activity, as shown here genetically and previously observed with chemical APC/C inhibitors (Lara-Gonzalez and Taylor, 2012; Zeng et al., 2010). Clearly, the interplay between SAC and APC/C activities is an important determinant of cellular behavior upon interference with mitosis, and, hence, therapeutic targeting of these key mitotic machines warrants further consideration.

## EXPERIMENTAL PROCEDURES

### Genome Editing

The *UBE2S*-, *UBE2C*-, and *MAD2*-knockout HCT116 cells were generated using the CRISPR/Cas9 technology (Cong et al., 2013; Mali et al., 2013). A donor plasmid containing a splice acceptor site and a resistance marker gene (puromycin, blasticidin, or hygromycin), flanked by homology arms, was co-transfected with pX330 (Cong et al., 2013), targeting the indicated DNA sequence within the respective gene. After drug resistance selection, cell colonies were screened by western blotting for the loss of the respective protein. Δ*UBE2S*Δ*UBE2C*-R cells were generated from Δ*UBE2S* cells by targeting *UBE2C* in the presence of reversine (0.5 μM). The following targeting sites were used: CCTCGGGCCCATCCCGGGTC for *UBE2S* (puromycin selection), CCACTAGCGTCGCCGCCGCC for *UBE2C* (blasticidin selection), and CACCCCTTACTTTCCGATAC and TACAGGACACGGTGTGACTG for *MAD2* (hygromycin selection). CCNB1 was endogenously tagged with Venus using recombinant adeno-associated virus (rAAV)-based genome engineering. The pAAV-CCNB1-Venus-targeting construct (Collin et al., 2013) was packed into rAAV particles in HEK293 cells as described previously (Berdougo et al., 2009). HCT116 WT, Δ*UBE2S*, Δ*UBE2C*, and Δ*UBE2S*Δ*UBE2C* #3 cells were infected with viral supernatant for 48 hr and expanded for 4 days post-infection. Mitotically enriched cells were collected and sorted using a BD FACSAria III sorter (BD Biosciences). Venus-positive single cells were individually sorted into single wells of a 96-well imaging plate; 12–16 days after the cell sorting, a screen for correctly targeted clones was performed using automated wide-field microscopy on an Olympus ScanR system (motorized IX81 microscope) with ScanR Image Acquisition Software, using an UPLSAPO 10×/0.4 numerical aperture (NA) dry objective and a MT20 Illumination system. Clones that exhibited Venus expression and the expected localization of CCBN1-Venus were retained and confirmed by western blotting.

### Live-Cell Imaging

Cells grown in an eight-well chamber (Ibidi) with L-15 medium supplemented with 10% fetal bovine serum (FBS) were mounted on a DeltaVision Elite microscope (GE Healthcare) and imaged using a 40× oil-immersion objective (1.35 NA, working distance [WD] 0.10). Images (differential interference contrast [DIC], yellow fluorescent protein [YFP], and red fluorescent protein [RFP]) were acquired continuously for 18–20 hr with 4- or 5-min intervals taking z stacks of 7 μm. Data analysis was performed using the softWorx software (GE Healthcare). ImageJ (NIH) was used to extract still images. The time from NEBD to anaphase onset was measured from the frame in which the nuclear envelope had disappeared to the frame in which the chromosomes had started to separate, as observed in DIC images. Each cell is represented in the graphs as a single circle. Red-filled circles indicate cells that did not exit mitosis within the time shown (e.g., due to end of filming) and, hence, represent a lower estimate.

CCNB1-Venus intensity was measured using the softWorx software (GE Healthcare) and time = 0 was set to two frames before initial CCNB1 degradation. The beginning of CCNB1 degradation was defined as a decrease in CCNB1-Venus intensity of >4%. Normalization to the beginning of CCNB1 degradation, rather than metaphase plate formation, was done to compare CCNB1 intensity decrease while eliminating mistakes of wrongly classifying a plate with subtle alignment errors as an error-free plate. The CCNB1 degradation curves depict cells from time = 0 to five frames after anaphase onset (the curve is depicted until all but one cell reached five frames after anaphase onset). To quantify differences in CCNB1 degradation, we calculated degradation rates around the maximum degradation. Background intensity was subtracted from the raw data and CCNB1 intensity was normalized to CCNB1 intensity at NEBD. The maximum difference in CCNB1 intensities between two time points was calculated to identify the time of maximal CCNB1 degradation. At least three time points were used, including the two time points at maximum degradation, to find the best fit to linear regression (highest R-squared value). The slope of the resulting linear regression gives the CCNB1 degradation rates around maximal CCNB1 degradation. Medians for mitotic timing (NEBD to anaphase onset and NEBD to metaphase or metaphase to anaphase onset) are depicted as red lines and stated on top of the graphs. The p values were calculated with Mann-Whitney tests. Means for CCNB1 degradation rates are depicted as a red line with SDs as vertical lines emerging from it, and stated on top of the graph. The p values were calculated with t tests. For statistical analysis, Prism (GraphPad software) was used; p values $\geq$ 0.01 were defined as non-significant.

### Antibodies Used for Western Blotting

The following antibodies were used: UBE2S (Cell Signaling Technology, 9630S or Abnova, PAB1701), Ubiquitin K11 linkage (Millipore, MABS107, clone 2A3/2E6), UBE2C (Boston Chemicals, A-650), MAD2 (Bethyl Laboraratories, A300-301A or homemade mouse antibody raised against full-length MAD2), CCNB1 (BD Pharmingen, 554176), MPS1 (Abcam, ab11108), Tubulin (Sigma-Aldrich, T5326 or Abcam, ab6160), GAPDH (Abcam, ab8245), UBE2D (Abcam, ab155088), Vinculin (Sigma, V9131), and GFP (Abcam, ab290).

### Cell Synchronization

To synchronize cells in mitosis, cells were cultured in thymidine- (2.5 mM) containing medium for 18 hr, released into medium without thymidine for 8 hr, and again cultured for 18 hr in medium containing thymidine (2.5 mM). After release from the second thymidine block into medium without thymidine, mitotic cells were harvested by shake-off at the mitotic peak (~10 hr after release from the thymidine block).

### Cellular DNA Analysis by Microscopy

Cells grown on coverslips were fixed with 4% paraformaldehyde (in PBS), stained with Hoechst (1 μg/ml in PBS), and mounted on coverslides using Vectashield as mounting medium. Microscopy was performed with a Leica wide-field system (DMI6000 B) using a 40× objective.

### Drug Treatments for Microscopy-Based Live-Cell Imaging Experiments

The APC/C inhibitor proTAME (Boston Biochem) was used at the indicated concentrations (5, 10, or 15 μM). To evaluate SAC activity, nocodazole was

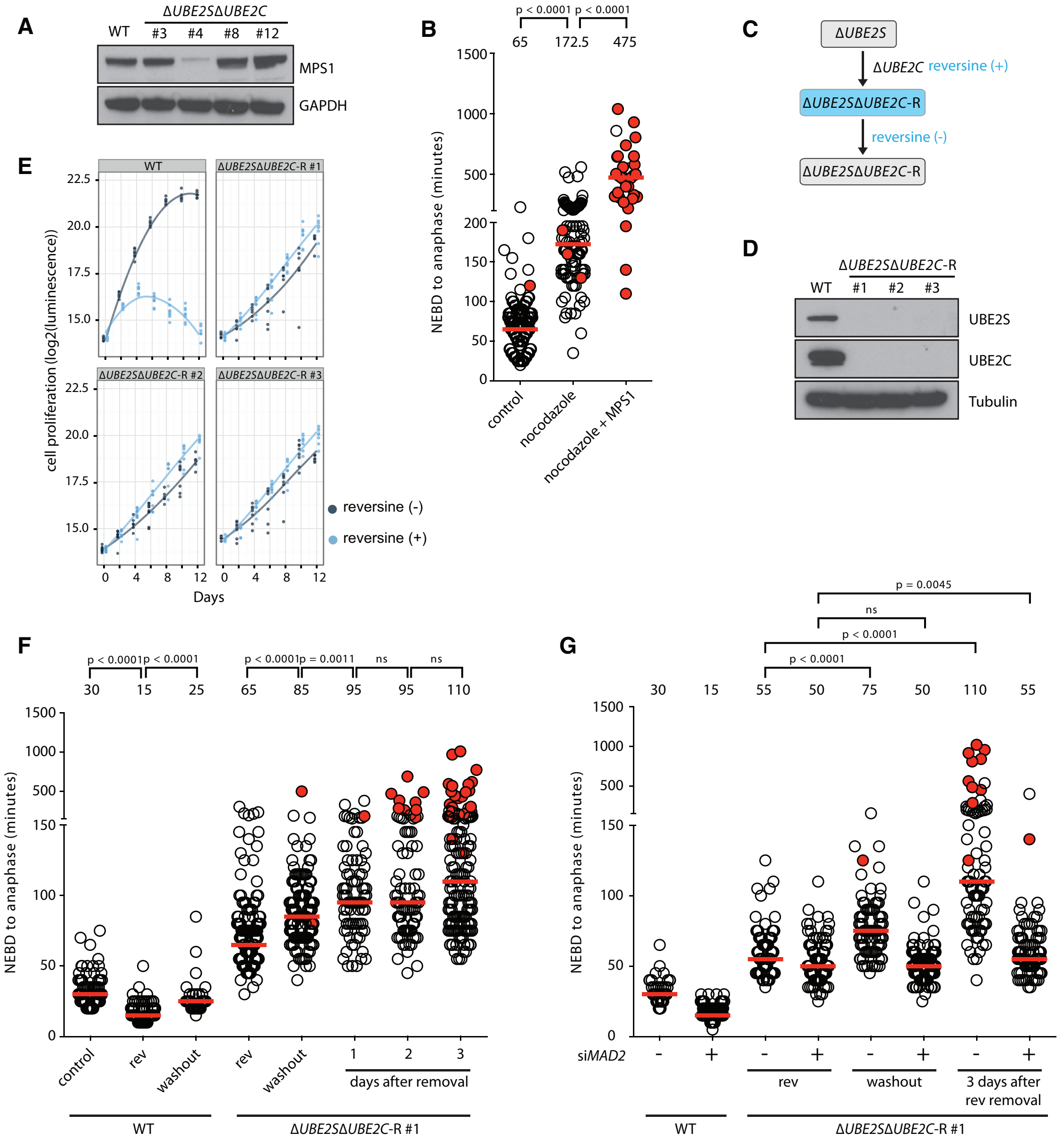


**Figure 5. Inhibition of the SAC Confers Beneficial Effects to Cells with Compromised APC/C Activity**

(A) Western blot analysis of MPS1 levels in WT and the indicated Δ*UBE2S*Δ*UBE2C* cell clones is shown.

(B) NEBD-to-anaphase onset timing in Δ*UBE2S*Δ*UBE2C* #4 in the presence or absence of nocodazole and upon TFP-MPS1 transfection in the presence of nocodazole. At least 30 cells were analyzed from three independent experiments. The median is depicted as a red line and noted above the data points. The p values for indicated conditions are stated on top.

(C) Schematic outline for the generation of Δ*UBE2S*Δ*UBE2C*-R cells. *UBE2C* was deleted in Δ*UBE2S* cells in the presence of reversine.

(D) Western blot analysis of UBE2S and UBE2C levels in WT and the indicated Δ*UBE2S*Δ*UBE2C*-R cell clones is shown.

(E) Growth analysis of the indicated cell lines in the presence or absence of 0.5 μM reversine. Cell proliferation was assessed for the indicated cells and times using a luminescence-based assay measuring ATP levels. A local regression curve is plotted for each cell line and condition based on at least six measurements per time point.

(F) Analysis of NEBD-to-anaphase onset timing by time-lapse microscopy (DIC). Each circle represents a single cell (red-filled circles indicate cells that did not exit mitosis within the stated time). WT cells were cultured with the following conditions: control, no reversine; rev, reversine added at the start of the experiment; and washout, cells cultured with reversine for 24 hr and reversine was removed just prior to the start of filming. Δ*UBE2S*Δ*UBE2C*-R cells were cultured in the following conditions: rev, cells were continuously cultured in reversine; days after washout, reversine was washed out from cells that were continuously cultured in reversine and filmed after the indicated number of days after reversine removal. The median NEBD-to-anaphase onset time is depicted as a red line and noted above the data points. At least 41 cells were analyzed from each cell line and condition. The p values were calculated with Mann-Whitney tests (ns, $p \geq 0.01$).

*(legend continued on next page)*

used at 30 ng/ml and reversine was used at 0.5 μM. Drugs were added to cells upon change to L-15 medium on the day of filming (1–2 hr prior to filming). To remove reversine from Δ*UBE2S*Δ*UBE2C*-R cells, cells were washed twice with PBS at indicated times and subsequently cultured in medium without reversine.

### Transient Transfections

For chromosome visualization, cells were treated with CellLight Histone H2B-RFP, BacMam 2.0 (Life Technologies) overnight. For reintroduction of MPS1, cells were transfected the day prior to live-cell imaging with TFP-MPS1 using Fugene 6. Transfection medium was removed and changed to L-15 medium on the day of filming.

## SUPPLEMENTAL INFORMATION

Supplemental Information includes Supplemental Experimental Procedures, five figures, and one table and can be found with this article online at http://dx.doi.org/10.1016/j.celrep.2016.01.060.

## AUTHOR CONTRIBUTIONS

T.W. and C.C. conceived the project. T.W. and M.S.Y.L. generated the data and, together with J.N. and C.C., they analyzed the data. T.N. helped with the data analysis. J.S. generated CCNB1-Venus-expressing cell lines. T.W. and C.C. wrote the manuscript. All authors contributed to designing the experiments and read and commented on the manuscript. C.C. coordinated the project.

## ACKNOWLEDGMENTS

We thank the members of our laboratories for their helpful discussions and Brian Tate Weinert for critically reading the manuscript. We acknowledge Philippe Collin and Jonathon Pines for kindly providing the pAAV-CCNB1-Venus-targeting construct, Bert Vogelstein for providing *TP53*-knockout HCT116 cells, and Gelo Dela Cruz from the flow cytometry core and Jutta Bulkescher and Jay Gopalakrishnan from the microscopy platform for their technical support. Work in the C.C. lab was supported by the Hallas Møller Investigator award from the Novo Nordisk Foundation, the European Research Council (ERC) consolidator grant from the European Commission, and the Sapere Aude program from the Danish Council for Independent Research. J.N. is supported by the Danish Cancer Society, the Lundbeck Foundation, and the Danish Council for Independent Research. The Novo Nordisk Foundation Center for Protein Research is supported financially by the Novo Nordisk Foundation (grant agreement NNF14CC0001).

Received: July 20, 2015
Revised: December 13, 2015
Accepted: January 1, 2016
Published: February 18, 2016

## REFERENCES

Abrieu, A., Magnaghi-Jaulin, L., Kahana, J.A., Peter, M., Castro, A., Vigneron, S., Lorca, T., Cleveland, D.W., and Labbé, J.C. (2001). Mps1 is a kinetochore-associated kinase essential for the vertebrate mitotic checkpoint. Cell *106*, 83–93.

Bastians, H., Topper, L.M., Gorbsky, G.L., and Ruderman, J.V. (1999). Cell cycle-regulated proteolysis of mitotic target proteins. Mol. Biol. Cell *10*, 3927–3941.

Ben-Eliezer, I., Pomerantz, Y., Galiani, D., Nevo, N., and Dekel, N. (2015). Appropriate expression of Ube2C and Ube2S controls the progression of the first meiotic division. FASEB J. *29*, 4670–4681.

Berdougo, E., Terret, M.E., and Jallepalli, P.V. (2009). Functional dissection of mitotic regulators through gene targeting in human somatic cells. Methods Mol. Biol. *545*, 21–37.

Buffin, E., Emre, D., and Karess, R.E. (2007). Flies without a spindle checkpoint. Nat. Cell Biol. *9*, 565–572.

Bunz, F., Dutriaux, A., Lengauer, C., Waldman, T., Zhou, S., Brown, J.P., Sedivy, J.M., Kinzler, K.W., and Vogelstein, B. (1998). Requirement for p53 and p21 to sustain G2 arrest after DNA damage. Science *282*, 1497–1501.

Burds, A.A., Lutum, A.S., and Sorger, P.K. (2005). Generating chromosome instability through the simultaneous deletion of Mad2 and p53. Proc. Natl. Acad. Sci. USA *102*, 11296–11301.

Collin, P., Nashchekina, O., Walker, R., and Pines, J. (2013). The spindle assembly checkpoint works like a rheostat rather than a toggle switch. Nat. Cell Biol. *15*, 1378–1385.

Cong, L., Ran, F.A., Cox, D., Lin, S., Barretto, R., Habib, N., Hsu, P.D., Wu, X., Jiang, W., Marraffini, L.A., and Zhang, F. (2013). Multiplex genome engineering using CRISPR/Cas systems. Science *339*, 819–823.

Dimova, N.V., Hathaway, N.A., Lee, B.H., Kirkpatrick, D.S., Berkowitz, M.L., Gygi, S.P., Finley, D., and King, R.W. (2012). APC/C-mediated multiple mono-ubiquitylation provides an alternative degradation signal for cyclin B1. Nat. Cell Biol. *14*, 168–176.

Fisk, H.A., and Winey, M. (2001). The mouse Mps1p-like kinase regulates centrosome duplication. Cell *106*, 95–104.

Furuta, T., Tuck, S., Kirchner, J., Koch, B., Auty, R., Kitagawa, R., Rose, A.M., and Greenstein, D. (2000). EMB-30: an APC4 homologue required for metaphase-to-anaphase transitions during meiosis and mitosis in Caenorhabditis elegans. Mol. Biol. Cell *11*, 1401–1419.

Garnett, M.J., Mansfeld, J., Godwin, C., Matsusaka, T., Wu, J., Russell, P., Pines, J., and Venkitaraman, A.R. (2009). UBE2S elongates ubiquitin chains on APC/C substrates to promote mitotic exit. Nat. Cell Biol. *11*, 1363–1369.

Hiruma, Y., Sacristan, C., Pachis, S.T., Adamopoulos, A., Kuijt, T., Ubbink, M., von Castelmur, E., Perrakis, A., and Kops, G.J. (2015). CELL DIVISION CYCLE. Competition between MPS1 and microtubules at kinetochores regulates spindle checkpoint signaling. Science *348*, 1264–1267.

Irniger, S., Piatti, S., Michaelis, C., and Nasmyth, K. (1995). Genes involved in sister chromatid separation are needed for B-type cyclin proteolysis in budding yeast. Cell *81*, 269–278.

Ji, Z., Gao, H., and Yu, H. (2015). CELL DIVISION CYCLE. Kinetochore attachment sensed by competitive Mps1 and microtubule binding to Ndc80C. Science *348*, 1260–1264.

Jin, L., Williamson, A., Banerjee, S., Philipp, I., and Rape, M. (2008). Mechanism of ubiquitin-chain formation by the human anaphase-promoting complex. Cell *133*, 653–665.

King, R.W., Peters, J.M., Tugendreich, S., Rolfe, M., Hieter, P., and Kirschner, M.W. (1995). A 20S complex containing CDC27 and CDC16 catalyzes the mitosis-specific conjugation of ubiquitin to cyclin B. Cell *81*, 279–288.

Kitagawa, R., Law, E., Tang, L., and Rose, A.M. (2002). The Cdc20 homolog, FZY-1, and its interacting protein, IFY-1, are required for proper chromosome segregation in Caenorhabditis elegans. Curr. Biol. *12*, 2118–2123.

Komander, D., and Rape, M. (2012). The ubiquitin code. Annu. Rev. Biochem. *81*, 203–229.

Kops, G.J., Weaver, B.A., and Cleveland, D.W. (2005). On the road to cancer: aneuploidy and the mitotic checkpoint. Nat. Rev. Cancer *5*, 773–785.

(G) NEBD-to-anaphase onset timing, analyzed as described in (F). 24 hr prior to filming, cells were treated with siRNAs targeting *MAD2* (+) or control (−) siRNA. Δ*UBE2S*Δ*UBE2C*-R cells were continuously cultured with 0.5 μM reversine (rev), or reversine was removed just prior to filming (washout) or 3 days before filming (3 days after rev removal). At least 81 cells were analyzed from two experiments (ns, $p \geq 0.01$).
See also Figure S5.

Lara-Gonzalez, P., and Taylor, S.S. (2012). Cohesion fatigue explains why pharmacological inhibition of the APC/C induces a spindle checkpoint-dependent mitotic arrest. PLoS ONE *7*, e49041.

Li, S.Z., Song, Y., Zhang, H.H., Jin, B.X., Liu, Y., Liu, W.B., Zhang, X.D., and Du, R.L. (2014). UbcH10 overexpression increases carcinogenesis and blocks ALLN susceptibility in colorectal cancer. Sci. Rep. *4*, 6910.

Liu, X., and Winey, M. (2012). The MPS1 family of protein kinases. Annu. Rev. Biochem. *81*, 561–585.

London, N., and Biggins, S. (2014). Signalling dynamics in the spindle checkpoint response. Nat. Rev. Mol. Cell Biol. *15*, 736–747.

Mali, P., Yang, L., Esvelt, K.M., Aach, J., Guell, M., DiCarlo, J.E., Norville, J.E., and Church, G.M. (2013). RNA-guided human genome engineering via Cas9. Science *339*, 823–826.

Matsumoto, M.L., Wickliffe, K.E., Dong, K.C., Yu, C., Bosanac, I., Bustos, D., Phu, L., Kirkpatrick, D.S., Hymowitz, S.G., Rape, M., et al. (2010). K11-linked polyubiquitination in cell cycle control revealed by a K11 linkage-specific antibody. Mol. Cell *39*, 477–484.

Meyer, H.J., and Rape, M. (2014). Enhanced protein degradation by branched ubiquitin chains. Cell *157*, 910–921.

Michel, L., Diaz-Rodriguez, E., Narayan, G., Hernando, E., Murty, V.V., and Benezra, R. (2004). Complete loss of the tumor suppressor MAD2 causes premature cyclin B degradation and mitotic failure in human somatic cells. Proc. Natl. Acad. Sci. USA *101*, 4459–4464.

Peters, J.M. (2006). The anaphase promoting complex/cyclosome: a machine designed to destroy. Nat. Rev. Mol. Cell Biol. *7*, 644–656.

Pines, J. (2011). Cubism and the cell cycle: the many faces of the APC/C. Nat. Rev. Mol. Cell Biol. *12*, 427–438.

Primorac, I., and Musacchio, A. (2013). Panta rhei: the APC/C at steady state. J. Cell Biol. *201*, 177–189.

Sackton, K.L., Dimova, N., Zeng, X., Tian, W., Zhang, M., Sackton, T.B., Meaders, J., Pfaff, K.L., Sigoillot, F., Yu, H., et al. (2014). Synergistic blockade of mitotic exit by two chemical inhibitors of the APC/C. Nature *514*, 646–649.

Santaguida, S., Tighe, A., D'Alise, A.M., Taylor, S.S., and Musacchio, A. (2010). Dissecting the role of MPS1 in chromosome biorientation and the spindle checkpoint through the small molecule inhibitor reversine. J. Cell Biol. *190*, 73–87.

Sivakumar, S., and Gorbsky, G.J. (2015). Spatiotemporal regulation of the anaphase-promoting complex in mitosis. Nat. Rev. Mol. Cell Biol. *16*, 82–94.

Sudakin, V., Ganoth, D., Dahan, A., Heller, H., Hershko, J., Luca, F.C., Ruderman, J.V., and Hershko, A. (1995). The cyclosome, a large complex containing cyclin-selective ubiquitin ligase activity, targets cyclins for destruction at the end of mitosis. Mol. Biol. Cell *6*, 185–197.

Summers, M.K., Pan, B., Mukhyala, K., and Jackson, P.K. (2008). The unique N terminus of the UbcH10 E2 enzyme controls the threshold for APC activation and enhances checkpoint regulation of the APC. Mol. Cell *31*, 544–556.

Tarailo, M., Kitagawa, R., and Rose, A.M. (2007). Suppressors of spindle checkpoint defect (such) mutants identify new mdf-1/MAD1 interactors in Caenorhabditis elegans. Genetics *175*, 1665–1679.

Wickliffe, K.E., Williamson, A., Meyer, H.J., Kelly, A., and Rape, M. (2011). K11-linked ubiquitin chains as novel regulators of cell division. Trends Cell Biol. *21*, 656–663.

Williamson, A., Wickliffe, K.E., Mellone, B.G., Song, L., Karpen, G.H., and Rape, M. (2009). Identification of a physiological E2 module for the human anaphase-promoting complex. Proc. Natl. Acad. Sci. USA *106*, 18213–18218.

Wu, T., Merbl, Y., Huo, Y., Gallop, J.L., Tzur, A., and Kirschner, M.W. (2010). UBE2S drives elongation of K11-linked ubiquitin chains by the anaphase-promoting complex. Proc. Natl. Acad. Sci. USA *107*, 1355–1360.

Yu, H., King, R.W., Peters, J.M., and Kirschner, M.W. (1996). Identification of a novel ubiquitin-conjugating enzyme involved in mitotic cyclin degradation. Curr. Biol. *6*, 455–466.

Zeng, X., Sigoillot, F., Gaur, S., Choi, S., Pfaff, K.L., Oh, D.C., Hathaway, N., Dimova, N., Cuny, G.D., and King, R.W. (2010). Pharmacologic inhibition of the anaphase-promoting complex induces a spindle checkpoint-dependent mitotic arrest in the absence of spindle damage. Cancer Cell *18*, 382–395.

Cell Reports, Volume *14*

## Supplemental Information

# The Spindle Assembly Checkpoint Is Not Essential for Viability of Human Cells with Genetically Lowered APC/C Activity

**Thomas Wild, Marie Sofie Yoo Larsen, Takeo Narita, Julie Schou, Jakob Nilsson, and Chunaram Choudhary**

Supplemental Information

# The spindle assembly checkpoint is not essential for viability of human cells with genetically lowered APC/C activity

Thomas Wild[1,*], Marie Sofie Yoo Larsen[2,*], Takeo Narita[1], Julie Schou[2], Jakob Nilsson[2,§], Chunaram Choudhary[1,§]

[1]Proteomics Program, the Novo Nordisk Foundation Center for Protein Research, Faculty of Health and Medical Sciences, University of Copenhagen, Blegdamsvej 3B, DK-2200 Copenhagen, Denmark

[2]Protein Signaling Program, the Novo Nordisk Foundation Center for Protein Research, Faculty of Health and Medical Sciences, University of Copenhagen, Blegdamsvej 3B, DK-2200 Copenhagen, Denmark

*These authors contributed equally to this work.

[§]Correspondence should be addressed to: jakob.nilsson@cpr.ku.dk, or chuna.choudhary@cpr.ku.dk

## Supplemental figures

# Figure S1

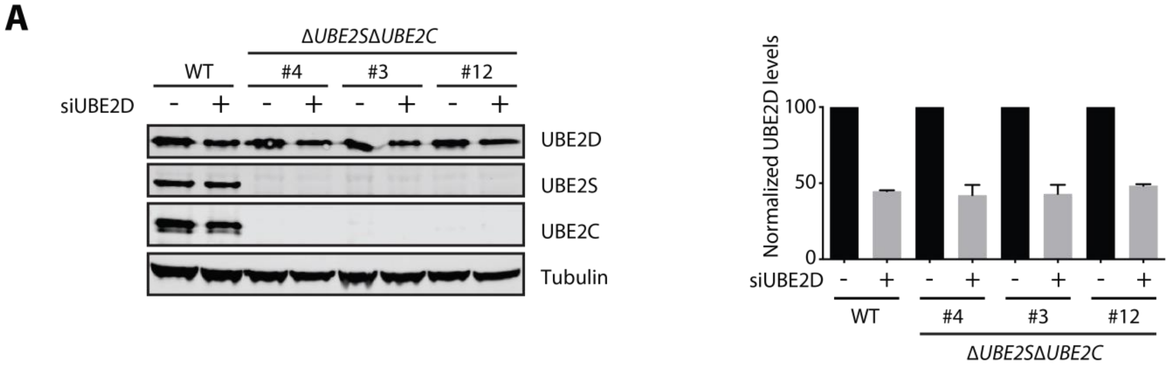


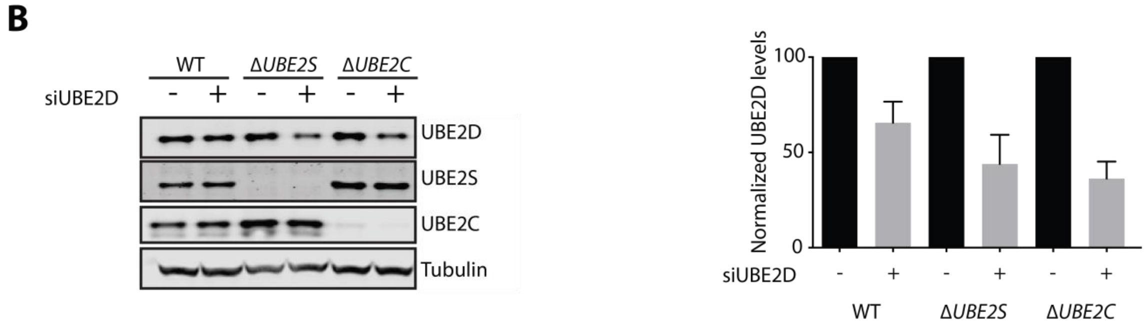


**Figure S1, related to Figure 2. Validation of UBE2D depletion by RNAi.**

- **A.** The left panel shows immunoblot-based validation of *UBE2D* knockdown in RNAi experiment shown in **Figure 2D**. Results from a representative experiment are shown. The bar chart (the right panel) shows *UBE2D* knockdown efficiency from two independent experiments. For each cell line, the normalized UBE2D levels show the ratio of UBE2D intensity over tubulin intensity. This ratio was set to 100% in the control siRNA condition and UBE2D levels in *UBE2D* siRNA-treated cells are shown relative to the control treated cells.
- **B.** Confirmation of *UBE2D* knockdown for the data shown in **Figure 2E**. The immunoblot (the left panel) shows results from a representative experiment. The bar chart (the right panel) shows knockdown efficiency from three independent experiments, the percent of UBE2D knockdown was determined as described in **Figure S1A**.

# Figure S2

**A**

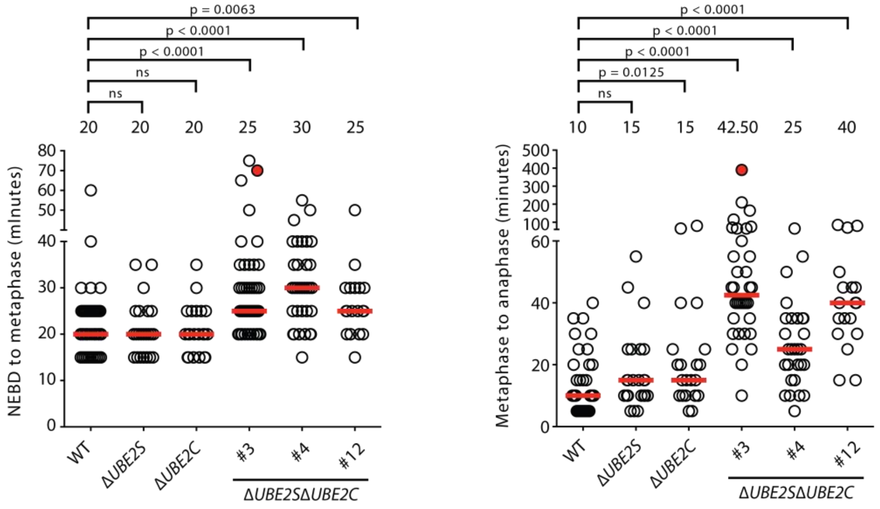
p = 0.0063
p < 0.0001
p < 0.0001
ns
ns
20 20 20 25 30 25
NEBD to metaphase (minutes)
WT
ΔUBE2S
ΔUBE2C
#3
#4
#12
ΔUBE2SΔUBE2C
p < 0.0001
p < 0.0001
p < 0.0001
p = 0.0125
ns
10 15 15 42.50 25 40
Metaphase to anaphase (minutes)

**B**

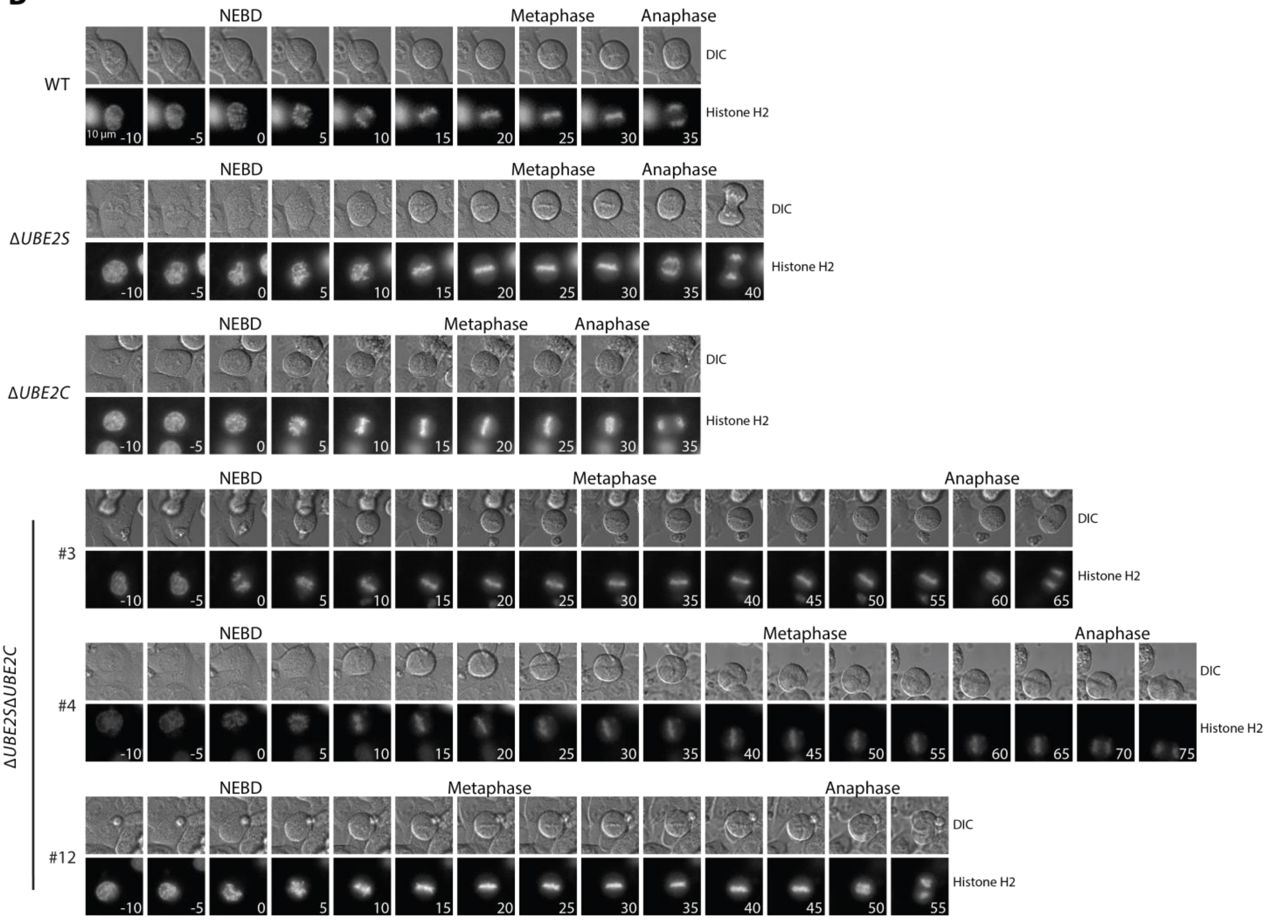
NEBD
Metaphase
Anaphase
DIC
Histone H2
WT
ΔUBE2S
ΔUBE2C
ΔUBE2SΔUBE2C
#3
#4
#12
10 µm

**Figure S2, related to Figure 2. Mitotic timing in ∆*UBE2S*, ∆*UBE2C*, and ∆*UBE2S*∆*UBE2C* cells.**

- **A.** Timing for NEBD to metaphase (left) and metaphase to anaphase onset (right) for the indicated cell lines. Cells were transiently transfected with histone H2B-RFP and analyzed by time-lapse microscopy. Each circle represents a single cell (red-filled circles indicate cells that did not exit mitosis within the stated time). The median time is depicted by a red line and noted on the top of the data points. For each cell line, at least 18 cells were analyzed from at least two independent experiments. P values were calculated with Mann-Whitney tests. ns = $p \geq 0.01$.
- **B.** Representative still images from live cell imaging experiment shown in **Figure S2A**. For ∆*UBE2S*∆*UBE2C* cells data are shown from three different cell clones.

# Figure S3

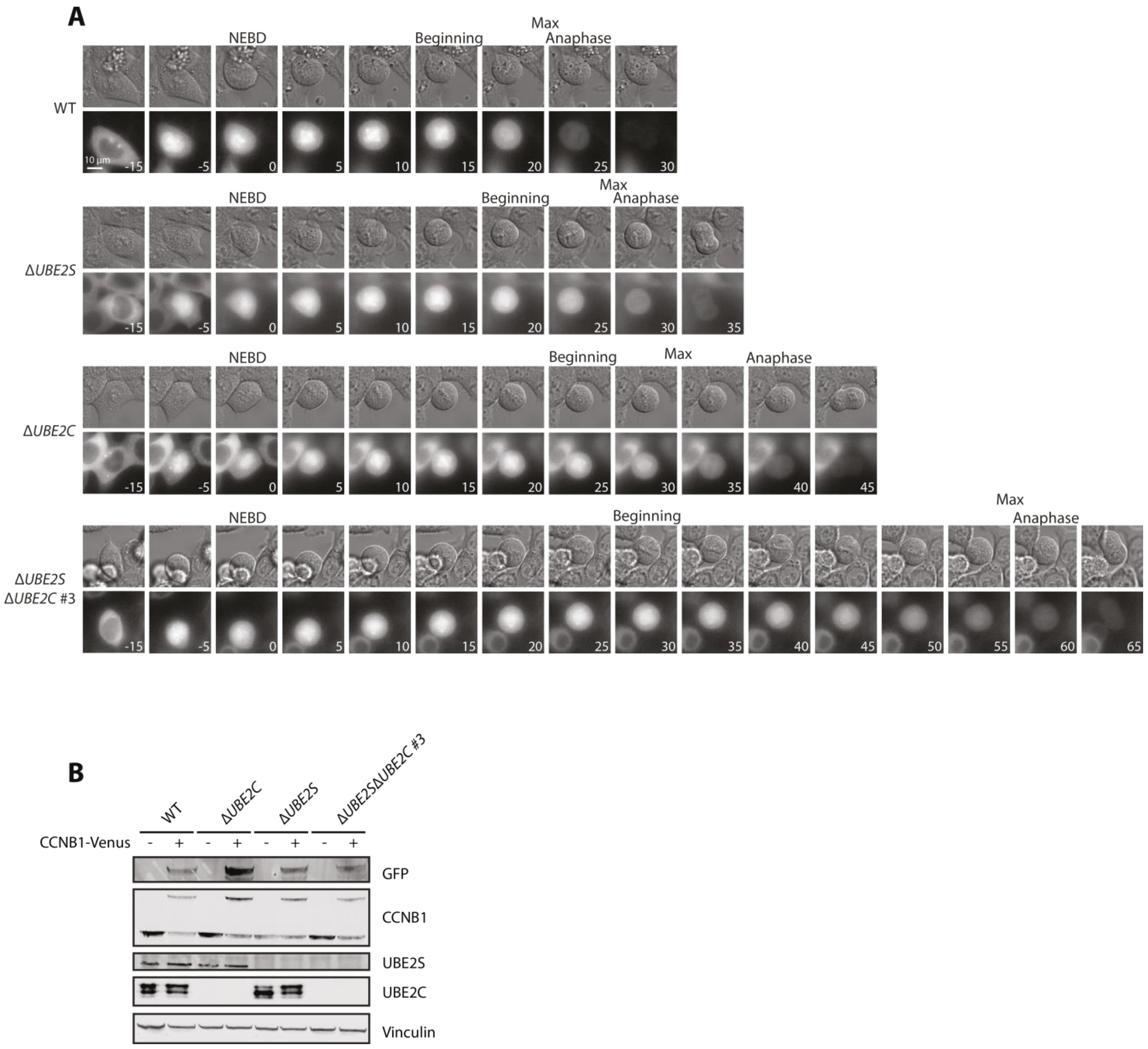


**Figure S3, related to Figure 2F,G. CCNB1 degradation in Δ*UBE2S*, Δ*UBE2C*, and Δ*UBE2S*Δ*UBE2C* cells.**

**A.** Representative still images from live cell imaging experiment shown in **Figure 2F**. Frames marking the beginning and maximal (max) CCNB1 degradation are labeled accordingly.

**B.** Western blot analysis of the indicated proteins in the analyzed cells. The figure confirms the expression of CCNB1-Venus (detected using a GFP antibody that also recognize Venus, and using a CCNB1 antibody). UBE2S and UBE2C immunoblots confirm the absence of these proteins in the respective knockout cells, and vinculin is shown as loading control.

# Figure S4

**A**

| Cell line | Reversine | R squared | LDT (h) | 95 % CI LDT | Intercept (ln) | 95 % CI ln | AUC | 95 % CI AUC |
|---|---|---|---|---|---|---|---|---|
| WT | - | 0.99 | 21.8 | 21.2 - 22.5 | 14.1 | 14.0 - 14.2 | 104.5 | 104.0 - 105.1 |
| *ΔUBE2S* | - | 0.99 | 24.0 | 23.3 - 24.8 | 14.5 | 14.3 - 14.6 | 104.8 | 104.5 - 105.1 |
| *ΔUBE2C* | - | 0.99 | 23.5 | 22.6 - 24.4 | 14.5 | 14.3 - 14.6 | 105.3 | 104.8 - 105.8 |
| *ΔUBE2SΔUBE2C* #3 | - | 0.95 | 37.0 | 34.1 - 40.4 | 14.4 | 14.2 - 14.6 | 98.2 | 97.5 - 99.0 |
| *ΔUBE2SΔUBE2C* #3 | + | 0.88 | 60.3 | 53.1 - 69.8 | 14.5 | 14.2 - 14.7 | 93.9 | 93.3 - 94.6 |
| *ΔUBE2SΔUBE2C* #4 | - | 0.96 | 34.8 | 32.5 - 37.4 | 14.5 | 14.3 - 14.6 | 99.3 | 98.7 - 99.9 |
| *ΔUBE2SΔUBE2C* #4 | + | 0.95 | 48.9 | 45.1 - 53.4 | 14.4 | 14.3 - 14.5 | 95.3 | 94.8 - 95.8 |
| *ΔUBE2SΔUBE2C* #8 | - | 0.99 | 33.3 | 32.4 - 34.3 | 14.3 | 14.3 - 14.4 | 99.0 | 98.8 - 99.3 |
| *ΔUBE2SΔUBE2C* #8 | + | 0.97 | 41.0 | 38.5 - 43.9 | 14.3 | 14.2 - 14.4 | 96.4 | 96.0 - 96.9 |
| *ΔUBE2SΔUBE2C* #12 | - | 0.99 | 32.6 | 31.6 - 33.7 | 14.2 | 14.2 - 14.3 | 98.7 | 98.4 - 99.0 |
| *ΔUBE2SΔUBE2C* #12 | + | 0.97 | 46.7 | 43.7 - 50.1 | 14.2 | 14.1 - 14.3 | 94.4 | 94.0 - 94.9 |

**B**

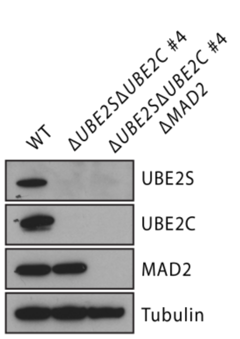


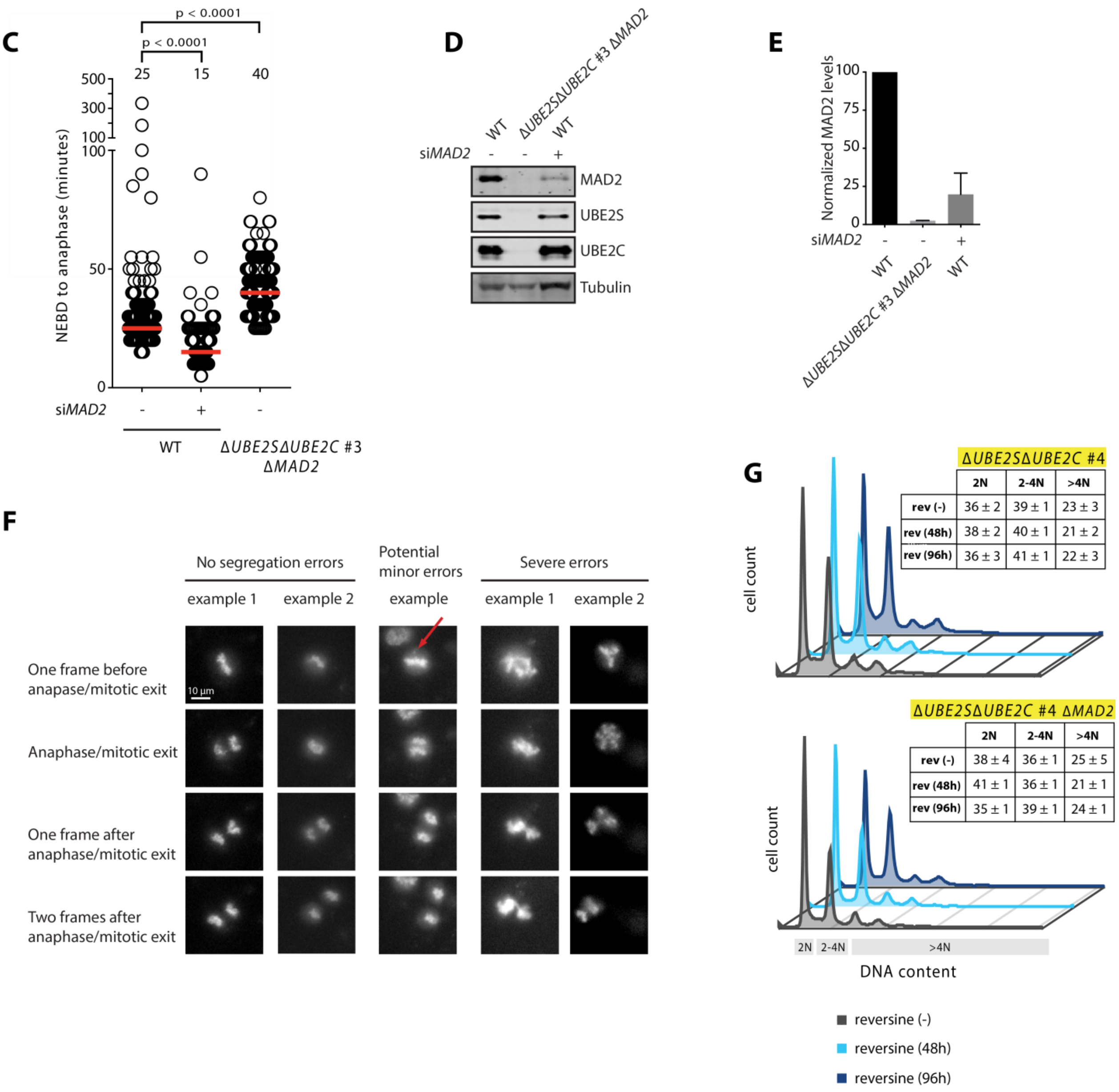

**Figure S4, related to Figure 3. The SAC is not essential in ∆*UBE2S*∆*UBE2C* cells.**

**A.** Calculation of cell doubling time from the data shown in **Figure 3B**. The indicated cell lines were cultured with or without reversine as indicated and cellular ATP content was measured every alternative day for up to 12 days using a luminescence-based assay. A linear regression model for luminescence intensity from day one to day six was used to calculate doubling times for the indicated cell lines and conditions. Cellular ATP content was used as a proxy for calculating cell proliferation; thus, LDT corresponds to cell doubling time. Abbreviations: luminescence doubling time (LDT), confidence interval (CI), intercept (In), area under the curve (AUC).

**B.** Western blot-based confirmation of MAD2 deletion in ∆*UBE2S*∆*UBE2C* #4 ∆*MAD2* cells, as control expression of MAD2 is show in the parental ∆*UBE2S*∆*UBE2C* clone #4 and WT cells.

**C.** Analysis of NEBD to anaphase onset timing in mitotic cells shown in **Figure 3D.** MAD2 was knocked down using siRNAs in WT cells and their mitotic phenotype was compared to ∆*UBE2S*∆*UBE2C* #3 ∆*MAD2* cells, in which MAD2 was completely deleted. At least 150 cells were analyzed from two independent experiments. The median is depicted as a red line and noted above the data points. P-values for indicated conditions are stated on top.

**D.** The immunoblot shows depletion of MAD2 in RNAi-based experiments presented in **Figure 3D**, the blot is representative from one of three experiments.

**E.** Quantification of MAD2 depletion in RNAi experiments shown in **Figure 3D and Figure S4C**, the data are from three independent experiments.

**F.** Representative still images from live cell imaging experiments shown in **Figure 3D**. When cells exited mitosis, the segregation of the chromosomes was categorized into three categories: no segregation errors, potential minor errors, or severe errors. The red arrow indicates a potential segregation error.

**G.** Analysis of cellular DNA content in ∆*UBE2S*∆*UBE2C* #4 and ∆*UBE2S*∆*UBE2C* #4 ∆*MAD2* cells upon reversine treatment. The cells were cultured in the indicated conditions and cellular DNA was stained with propidium iodide and cells were analyzed by flow cytometry.

# Figure S5

**A**

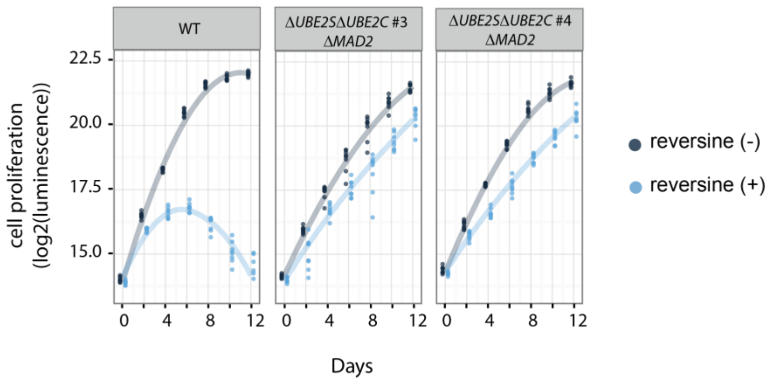


**B**

| Cell line | Reversine | R squared | LDT (h) | 95 % CI LDT | Intercept (In) | 95 % CI In | AUC | 95 % CI AUC |
|---|---|---|---|---|---|---|---|---|
| WT | - | 0.99 | 22.6 | 22.0 - 23.3 | 14.1 | 14.0 - 14.3 | 103.9 | 103.7 - 104.2 |
| Δ*UBE2S*Δ*UBE2C* #3 Δ*MAD2* | - | 0.97 | 31.9 | 29.9 - 34.1 | 14.3 | 14.1 - 14.4 | 99.5 | 98.9 - 100.1 |
| Δ*UBE2S*Δ*UBE2C* #3 Δ*MAD2* | + | 0.92 | 38.9 | 35.1 - 43.5 | 14.0 | 13.8 - 14.2 | 94.9 | 94.0 - 95.8 |
| Δ*UBE2S*Δ*UBE2C* #4 Δ*MAD2* | - | 0.99 | 29.6 | 28.8 - 30.4 | 14.4 | 14.4 - 14.5 | 101.4 | 101.1 - 101.6 |
| Δ*UBE2S*Δ*UBE2C* #4 Δ*MAD2* | + | 0.96 | 43.3 | 40.5 - 46.6 | 14.4 | 14.3 - 14.5 | 96.5 | 96.0 - 96.9 |

**Figure S5, related to Figure 5. Assessment of SAC function in Δ*UBE2S*Δ*UBE2C*Δ*MAD2* cells.**

**A.** Growth analysis of Δ*UBE2S*Δ*UBE2C*Δ*MAD2* cells (clone #3 and #4) in the presence or absence of 0.5 µM reversine, WT cells were used as reference.

**B.** Doubling times for the indicated cell lines and condition. The doubling time was calculated as described in **Figure S4A**. Abbreviations: luminescence doubling time (LDT), confidence interval (CI), intercept (In), area under the curve (AUC).

**Legend for Supplemental Table S1. SILAC-based proteomic analysis of the knockout cells.** SILAC-based quantification of changes in protein levels in knockout cells compared to WT cells. The spreadsheet indicates the relative changes in protein expression in the indicated knockout cells compared to WT cells.

## Supplemental methods

### RNA-mediated interference

Cells were transfected with siRNAs (final concentration of 50 nM) using RNAiMAX (Lipofectamine®) the day before the start of live cell imaging. For depletion of UBE2D, a previously described pool of siRNAs was used (Dynek et al., 2010). For MAD2 depletion, the following siRNA oligo was used: 5'-GGAAGAGUCGGGACCACAG-3'. As a negative control, a siRNA targeting luciferase (Sigma, VC300B2) was used. All siRNAs were obtained from Sigma. To evaluate knockdown efficiencies, the expression of target proteins was analyzed by Western blotting. For quantification, the blots were scanned using the Odyssey Sa imaging system (Li-Cor) and quantification was carried out using the Odyssey Sa Application software (Li-Cor) with IRDye 800 or 680 secondary antibodies (Li-Cor).

### Cell proliferation assay

For each cell line and condition, 400 cells were seeded in octuplicate in a 96-well plate. Cells were grown in the absence or presence of reversine (0.5 μM) and the medium was changed every second day.  Cell viability was assessed at different time points (day 0 = 12 hours after seeding, before addition of reversine) using the CellTiter-Glo® Luminescent Cell Viability Assay (Promega) according to the manufacturer`s protocol, measuring luminescence with a Varioskan Flash (Thermo Scientific). The local regression curves were plotted using geom_line() function in R package "ggplot2" (Wickham, 2009) with the following parameters: stat = "smooth", method = "loess", span = 2. Based on the observed luminescence curves, we used data from day zero to six to calculate growth rates for all cell lines. For this, log-transformed luminescence intensities were fitted to a linear regression model. The quality of the linear fit is given by R squared and the luminescence doubling time (LDT) was calculated from the slope of the linear fit. The 95% confidence intervals were calculated for the LDT, intercept and AUC. The linear regression analysis was performed by the lm function in R, adjusted R squared values were calculated using R base function lm() with Wherry's equation (Team, 2015) and the AUC was calculated with the grofit package (Kahm et al., 2010).

### MS-based analysis of proteome changes

For SILAC-based quantification of proteins (Ong et al., 2002), wild-type cells were grown in medium containing natural variants of L-arginine and L-lysine ($Arg^0/Lys^0$) and knockout cell lines in medium containing heavy isotope labeled variants of L-arginine and L-lysine ($Arg^{10}/Lys^8$). Cells were lysed in RIPA buffer (50 mM Tris-HCl pH 7.5, 150 mM NaCl, 1% Nonidet P-40, 0.1% sodium-dodecyl-sulfate, 1 mM EDTA) supplemented with protease inhibitors (Complete protease inhibitor mixture tablets, Roche Diagnostics). Lysates were incubated for 10 min on ice and cleared by centrifugation at 16,000 × g. An equal amount of protein from the "light" (wild-type) and the "heavy" (respective knockout cells) SILAC condition was mixed and SDS sample buffer added. Samples in SDS sample buffer were incubated with dithiothreitol (10 mM) for 10 min at 70 °C and alkylated with chloroacetamide (5.5 mM) for 60 min at 25 °C. Proteins were separated by SDS-PAGE using a 4%-12% gradient gel, visualized with colloidal coomassie blue stain, and digested using in-gel digestion method (Shevchenko et al., 2006). Gel lanes were cut into ten separate fractions and each gel piece was further sliced into small pieces (~1mm). Gel pieces were destained with 50% ethanol in 25 mM ammonium bicarbonate (pH 8.0) and dehydrated with 100% ethanol. Trypsin (in 25 mM ammonium bicarbonate pH 8.0) was added and incubated overnight. The trypsin digestion was stopped by addition of trifluoroacetic acid (0.5% final concentration) and peptides were eluted from the gel pieces by stepwise increase in acetonitrile concentration (to 100% final). Subsequently, acetonitrile was removed by centrifugal evaporation and peptides were purified by C18 reversed-phase packed Stage-Tips (Rappsilber et al., 2007). In a complementary approach cells were lysed in denaturing buffer (6 M urea, 2 M thiourea, 10 mM HEPES pH 8.0) and equal amounts of protein from "light" and "heavy" SILAC states were mixed. Protein were reduced with dithiothreitol (1 mM), alkylated with chloroacetamide (5.5 mM), four-fold-diluted with water and digested with trypsin overnight. The protein digestion was stopped by addition of trifluoroacetic acid (0.5% final concentration) and centrifuged at 16,000 × g for 10 min. Cleared peptides were fractionated by micro-column-based strong-cation exchange chromatography (SCX) into six fractions and cleaned by C18 reversed-phase packed Stage-Tips.

Peptide fractions were analyzed on a quadrupole Orbitrap (Q-Exactive, Thermo Scientific) mass spectrometer equipped with a nanoflow HPLC system (Thermo Scientific). Peptide samples were loaded onto C18 reversed-phase columns and eluted with a linear gradient from 8 to 40% acetonitrile containing 0.5% acetic acid in 2-3 hours. The Q-Exactive was operated in the data-dependent mode automatically switching between MS and MS-MS. Survey full-scan MS spectra (m/z 300–1700) were acquired in the Orbitrap. The 10 most intense ions were sequentially isolated

and fragmented by higher-energy C-trap dissociation (HCD). Peptides with unassigned charge states, as well as peptides with charge state less than +2 were excluded from fragmentation. Fragment spectra were acquired in the Orbitrap mass analyzer.

Raw MS data were analyzed by the MaxQuant software (version 1.5.0.38) (Cox and Mann, 2008). Mass spectra were searched against protein sequences from the UniProt knowledge base (downloaded January 2014) using the Andromeda search engine (Cox et al., 2011). Spectra were searched with a mass tolerance of 6 ppm for precursor ions, 20 ppm for fragment ions, strict trypsin specificity and allowing up to two missed cleavage sites. Cysteine carbamido methylation was searched as a fixed modification, whereas amino-terminal protein acetylation and methionine oxidation were searched as variable modifications. A false discovery rate of less than one percent was achieved using target-decoy search strategy (Elias and Gygi, 2007) and a posterior error probability filter. The raw data and MaxQuant output files have been deposited to the ProteomeXchange Consortium (http://proteomecentral.proteomexchange.org) via the PRIDE partner repository (Vizcaino et al., 2016) with the dataset identifier PXD003443.

**Cell cycle profile analysis**

Equal amounts of cells were seeded into three 6 cm dishes (20,000 cells per dish) and cultured for 12 hours before the start of the experiment. The experiment was performed by culturing cells in three different conditions- untreated, 48 hours reversine, and 96 hours reversine. The untreated cells were cultured during the entire experiment in medium without reversine and medium was changed after 48 hours. For the 48 hours reversine sample, cells were cultured in medium without reversine for the first 48 hours, and for the last 48 hours with medium containing reversine (0.5 μM). For the 96 hours reversine sample, cells were cultured during the entire experiment in medium with reversine (0.5 μM) and medium was changed after 48 hours. Cells from the three conditions (untreated, 48 hours reversine, 96 hours reversine) were trypsinized, washed twice in cold PBS and resuspended in 500 μl ice-cold PBS. Cells were permeablized by addition of 500 μl ice-cold ethanol, incubated for 45 min on ice, pelleted by centrifugation and washed with cold PBS. The cell pellet was resuspended in propidium iodide (PI) staining solution (10 μg/ml PI in PBS) supplemented with RNAse (25 μg/ml) and incubated for 30 min at 37 °C. Samples were analyzed with flow cytometry using a BD FACSCalibur (BD Biosciences), and the data were analyzed and visualized using the FlowJo software. To determine cell populations with different DNA content

(i.e. 2N, 2N-4N (including 4N), and >4N cell populations) gates were manually set for the wildtype untreated sample and applied to all samples of the same experiment.

## **Supplemental references**


Cox, J., and Mann, M. (2008). MaxQuant enables high peptide identification rates, individualized p.p.b.-range mass accuracies and proteome-wide protein quantification. Nature biotechnology *26*, 1367-1372.

Cox, J., Neuhauser, N., Michalski, A., Scheltema, R.A., Olsen, J.V., and Mann, M. (2011). Andromeda: a peptide search engine integrated into the MaxQuant environment. Journal of proteome research *10*, 1794-1805.

Dynek, J.N., Goncharov, T., Dueber, E.C., Fedorova, A.V., Izrael-Tomasevic, A., Phu, L., Helgason, E., Fairbrother, W.J., Deshayes, K., Kirkpatrick, D.S.*, et al.* (2010). c-IAP1 and UbcH5 promote K11-linked polyubiquitination of RIP1 in TNF signalling. Embo J *29*, 4198-4209.

Elias, J.E., and Gygi, S.P. (2007). Target-decoy search strategy for increased confidence in large-scale protein identifications by mass spectrometry. Nature methods *4*, 207-214.

Kahm, M., Hasenbrink, G., Lichtenberg-Frate, H., Ludwig, J., and Kschischo, M. (2010). grofit: Fitting Biological Growth Curves with R. Journal of Statistical Software. Journal of Statistical Software *33*, 1-21.

Ong, S.E., Blagoev, B., Kratchmarova, I., Kristensen, D.B., Steen, H., Pandey, A., and Mann, M. (2002). Stable isotope labeling by amino acids in cell culture, SILAC, as a simple and accurate approach to expression proteomics. Mol Cell Proteomics *1*, 376-386.

Rappsilber, J., Mann, M., and Ishihama, Y. (2007). Protocol for micro-purification, enrichment, pre-fractionation and storage of peptides for proteomics using StageTips. Nature protocols *2*, 1896-1906.

Shevchenko, A., Tomas, H., Havlis, J., Olsen, J.V., and Mann, M. (2006). In-gel digestion for mass spectrometric characterization of proteins and proteomes. Nature protocols *1*, 2856-2860.

Team, R.C. (2015). A language and environment for statistical computing.

Vizcaino, J.A., Csordas, A., Del-Toro, N., Dianes, J.A., Griss, J., Lavidas, I., Mayer, G., Perez-Riverol, Y., Reisinger, F., Ternent, T., *et al.* (2016). 2016 update of the PRIDE database and its related tools. Nucleic acids research *44*, D447-456.

Wickham, H. (2009). ggplot2: elegant graphics for data analysis (Springer New York).
